# Disrupting the ArcA Regulatory Network Amplifies the Fitness Cost of Tetracycline Resistance in Escherichia coli

**DOI:** 10.1128/msystems.00904-22

**Published:** 2022-12-20

**Authors:** Mario L. Arrieta-Ortiz, Min Pan, Amardeep Kaur, Evan Pepper-Tunick, Vivek Srinivas, Ananya Dash, Selva Rupa Christinal Immanuel, Aaron N. Brooks, Tyson R. Shepherd, Nitin S. Baliga

**Affiliations:** a Institute for Systems Biology, Seattle, Washington, USA; b Molecular Engineering Sciences Institute, University of Washington, Seattle, Washington, USA; c Inscripta Inc, Boulder, Colorado, USA; d Department of Biology, University of Washington, Seattle, Washington, USA; e Molecular and Cellular Biology Program, University of Washington, Seattle, Washington, USA; f Lawrence Berkeley National Lab, Berkeley, California, USA; g Department of Microbiology, University of Washington, Seattle Washington, USA; Génomique Métabolique, Genoscope, Institut François Jacob, CEA, CNRS, Université d’Évry, Université Paris-Saclay

**Keywords:** ArcA, *Escherichia coli*, antibiotic resistance, compensatory mechanism, global regulatory networks, systems biology, tetracycline

## Abstract

There is an urgent need for strategies to discover secondary drugs to prevent or disrupt antimicrobial resistance (AMR), which is causing >700,000 deaths annually. Here, we demonstrate that tetracycline-resistant (Tet^R^) Escherichia coli undergoes global transcriptional and metabolic remodeling, including downregulation of tricarboxylic acid cycle and disruption of redox homeostasis, to support consumption of the proton motive force for tetracycline efflux. Using a pooled genome-wide library of single-gene deletion strains, at least 308 genes, including four transcriptional regulators identified by our network analysis, were confirmed as essential for restoring the fitness of Tet^R^ E. coli during treatment with tetracycline. Targeted knockout of ArcA, identified by network analysis as a master regulator of this new compensatory physiological state, significantly compromised fitness of Tet^R^ E. coli during tetracycline treatment. A drug, sertraline, which generated a similar metabolome profile as the *arcA* knockout strain, also resensitized Tet^R^ E. coli to tetracycline. We discovered that the potentiating effect of sertraline was eliminated upon knocking out *arcA*, demonstrating that the mechanism of potential synergy was through action of sertraline on the tetracycline-induced ArcA network in the Tet^R^ strain. Our findings demonstrate that therapies that target mechanistic drivers of compensatory physiological states could resensitize AMR pathogens to lost antibiotics.

**IMPORTANCE** Antimicrobial resistance (AMR) is projected to be the cause of >10 million deaths annually by 2050. While efforts to find new potent antibiotics are effective, they are expensive and outpaced by the rate at which new resistant strains emerge. There is desperate need for a rational approach to accelerate the discovery of drugs and drug combinations that effectively clear AMR pathogens and even prevent the emergence of new resistant strains. Using tetracycline-resistant (Tet^R^) Escherichia coli, we demonstrate that gaining resistance is accompanied by loss of fitness, which is restored by compensatory physiological changes. We demonstrate that transcriptional regulators of the compensatory physiologic state are promising drug targets because their disruption increases the susceptibility of Tet^R^ E. coli to tetracycline. Thus, we describe a generalizable systems biology approach to identify new vulnerabilities within AMR strains to rationally accelerate the discovery of therapeutics that extend the life span of existing antibiotics.

## INTRODUCTION

Antimicrobial resistance (AMR) is the ability of a bacterium to withstand growth inhibition and killing by high doses of an antibiotic (1, 2). The problem of AMR has emerged from the overprescription and overuse of antibiotics (3–5), accumulation of antibiotics in the natural environment (6), antibiotic-induced increased mutation rates (7, 8), horizontal transfer of resistance-conferring genes (3), and poor infection-control strategies (9). As a result, infections by pathogenic AMR strains are rapidly growing and projected to cause ~10 million deaths/year by 2050 (4). Sadly, consistent with this prediction, ~5 million deaths in 2019 were associated with infections caused by AMR strains of bacterial pathogens (10). While health policies to regulate antibiotic use (11) and programs to ensure patient compliance with completing prescribed antibiotic regimens are effective (12), these efforts are expensive, laborious, and face implementation challenges around the world (13). Similarly, efforts to find new potent antibiotics are effective (14) but also expensive and being outpaced by the rate at which new resistant strains are emerging (15).

A solution to tackling AMR might be in the observation that gaining resistance to an antibiotic is typically associated with loss of fitness (3), which can be restored through compensatory mutations (3) that cause changes in regulation and metabolism (16–18). For example, Pseudomonas aeruginosa upregulates anaerobic nitrate respiration to quench intracellular protons and compensate for loss of fitness due to efflux-mediated resistance (19). Similarly, Mycobacterium smegmatis transcriptionally upregulates the rRNA methylase TlyA to restore fitness upon gaining resistance to capreomycin (20). Molecules that target new vulnerabilities within compensatory mechanisms of antibiotic resistance could enable the recovery of “lost” antibiotics and broaden the life span of new antibiotics (21, 22). The ability of metabolite supplementation to resensitize resistant pathogens to diverse antibiotics, including aminoglycosides (23), chloramphenicol, and streptomycin (24), lends credibility to this idea. However, to implement such a strategy at scale, we need to develop methodology to discover the mechanistic driver(s) of fitness-restoring compensatory changes in AMR strains, confirm with targeted genetic perturbations that these mechanistic drivers do indeed represent new vulnerabilities, and use a rational approach to find molecules that could disrupt the compensatory mechanism (25).

Here, we have developed a systems approach to discover and target mechanistic drivers of the compensatory physiologic state of tetracycline-resistant (here called Tet^R^) Escherichia coli. Discovered in 1947, tetracyclines are protein synthesis inhibitors that act by binding to the 30S ribosomal subunit (26, 27). Tetracyclines were rapidly adopted in the clinic due to their broad spectrum efficacy (26, 27) and continue to be used widely in animal farming (28). Resistance to tetracyclines emerged a few years later in 1953 and progressively reduced their effectiveness (26, 27). The primary mechanisms of tetracycline resistance are (i) through active extrusion by efflux pumps; (ii) gain of mutations that disrupt interaction with the target; and (iii) enzymatic inactivation, e.g., by TetX (26, 27). Previous attempts to counteract tetracycline resistance in E. coli have focused on potential efflux pump inhibitors (29, 30).

We have discovered that when E. coli gains resistance to tetracycline through AcrAB-mediated efflux, a global shift in metabolism to a fermentative state is required to restore fitness of the resistant strain in the presence of tetracycline. The regulatory network that mechanistically drives this global metabolic reprogramming in the Tet^R^ strain is comprised of at least 25 transcription factors (TFs) that directly regulate 279 genes. Interestingly, 209 of the 279 genes are differentially regulated by 15 TFs in the presence of tetracycline, suggesting that increased activity of the AcrAB efflux pump causally alters the activity of these regulators. Using a pooled barcoded library of CRISPR-generated knockout strains, we performed a genome-wide fitness screen that uncovered 308 genes essential for restoring fitness of Tet^R^ E. coli during treatment with tetracycline. In fact, the fitness screen validated mechanistic predictions from our network-based strategy that four TFs (ArcA, CytR, PhoP, and RpoS) contributed to the compensatory physiologic state required for restoring the fitness of Tet^R^ E. coli in the presence of tetracycline. Further, the fitness screen also confirmed that, as predicted, tetracycline treatment drove Tet^R^ E. coli from aerobic respiration toward a fermentative physiologic state. Targeted knockout of *arcA*, a master regulator of this network, further confirmed its role in restoring fitness of the Tet^R^ strain. We discovered that the drug sertraline, which generated a similar metabolome profile as the *arcA* knockout, potentiated the bacteriostatic effect of tetracycline on the Tet^R^ strain, but not the wild-type strain, on which the effect was additive. We also show that deleting *arcA* abolished the potentiating effect of sertraline, demonstrating that the mechanism of its potential synergy with tetracycline was through its action on the tetracycline-induced and ArcA-regulated network. We discuss these results from the perspective of formulating a multidrug regimen using a network-based approach to recover lost antibiotics and prolong the utility of new antibiotics.

## RESULTS

### A novel physiological state underlies tetracycline resistance in E. coli.

To identify mutations that may contribute to the tetracycline resistance phenotype of Tet^R^ E. coli, we resequenced and compared the genomes of the tetracycline-susceptible wild-type (MG1655, here called “WT”) and laboratory-evolved Tet^R^ strains of E. coli (31, 32). We discovered that gaining tetracycline resistance could be due to mutations in *acrB* and *acrR* genes, which were independently reported by Hoeksema et al. (33) (complete list of mutations shown in Table S1). AcrR is a transcriptional repressor of the *acrAB* operon, and mutations in this gene are consistent with upregulation of the efflux pump (see below) (33). Interestingly, we also identified an in-frame deletion in *mlaA*, which encodes a component of the system for maintenance of lipid asymmetry (34). He et al. have recently associated this deletion in the *mlaA* gene with resistance to tigecycline (a glycylcycline, a tetracycline derivative [35]), even in the absence of *acrAB* (36). Thus, multiple mechanisms may simultaneously contribute to the resistance phenotype.

10.1128/msystems.00904-22.7 TABLE S1 Mutations identified in the Tet^R^ strain. Download Table S1, DOCX file, 0.02 MB. Copyright © 2022 Arrieta-Ortiz et al. 2022 Arrieta-Ortiz et al. https://creativecommons.org/licenses/by/4.0/ This content is distributed under the terms of the Creative Commons Attribution 4.0 International license.

**FIG 1:**
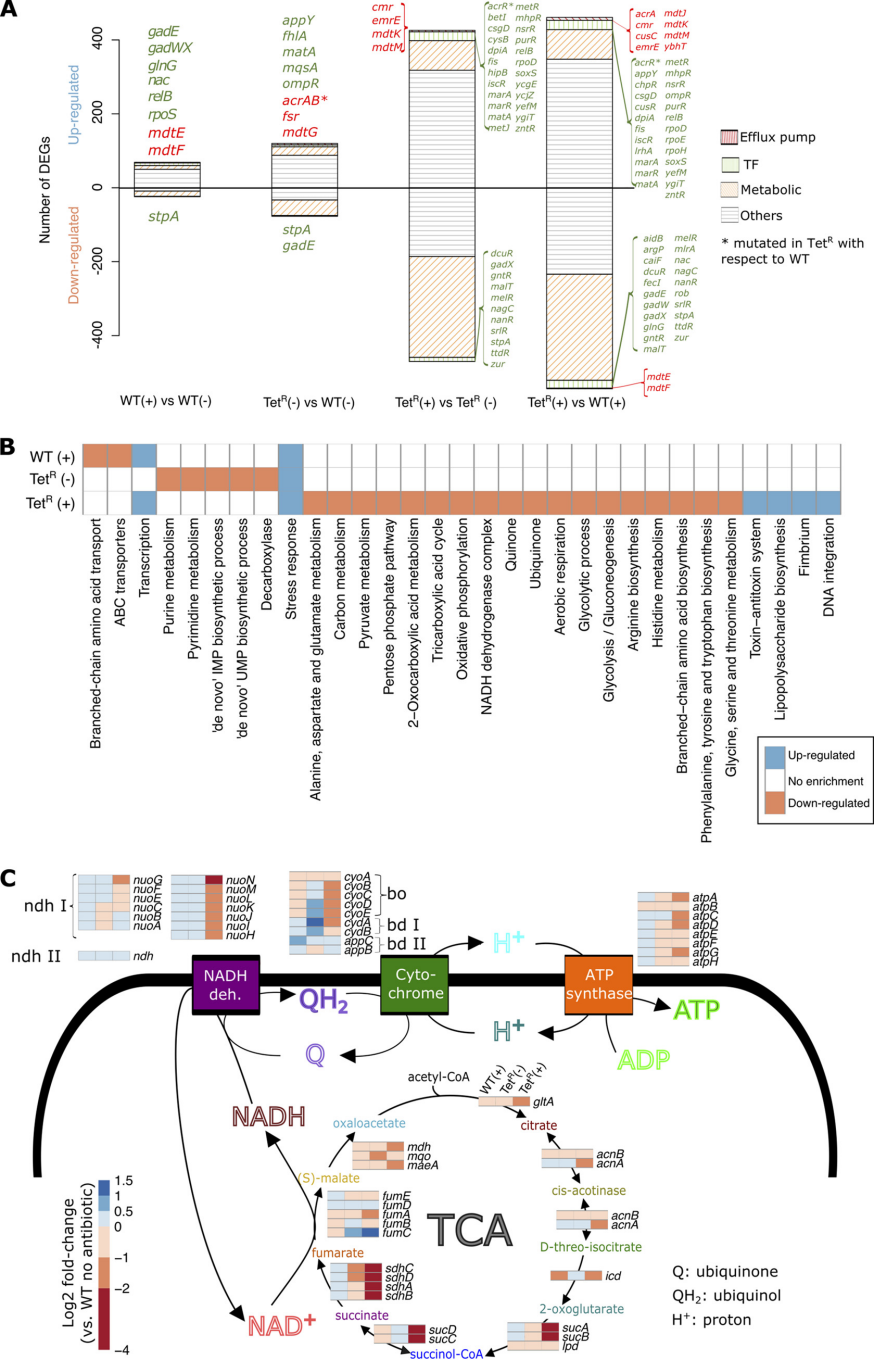
Transcriptional and metabolic remodeling accompanying gain of tetracycline resistance in E. coli. (A) Comparison of transcriptomes of Tet^R^ and parental WT (MG1655) strains in the presence (+) and absence of tetracycline (−). Tetracycline concentrations used for wild-type (WT) and Tet^R^ strains were 0.25 and 16 μg/mL, respectively. Transcriptomics data were sourced from Händel et al. (32). Differentially expressed genes (adjusted *P* value < 0.05 and absolute log_2_ fold change > 1) were classified as efflux pump-related (compiled from the EcoCyc database and available literature) (53, 102), transcription factors (TFs) (based on the transcriptional regulatory network compiled from the RegulonDB database), or metabolism-related (based on the iJO1366 metabolic model of E. coli) (40, 102, 103). Unassigned genes were grouped in the “Others” category. Differentially expressed TFs and efflux pump genes are listed in green and red type, respectively. (B) Heat map with functional enrichment information of the set of genes significantly up- and downregulated in the WT strain in the presence of tetracycline and the Tet^R^ strain without and with tetracycline with respect to the WT strain in antibiotic-free condition. Due to space constraints and functional terms redundancy, only a subset of functional terms are displayed (full list on Table S2). (C) Fold change profiles (with respect to the WT strain in antibiotic free condition) of genes related to the tricarboxylic acid (TCA) cycle, the electron transport chain (i.e., NADH dehydrogenases [NADH deh.], and cytochromes), and ATP synthase. Pathways of interest and associated genes were compiled from the EcoCyc database (102) and available literature (104). DEG, differentially expressed gene.

To characterize direct and compensatory physiological changes triggered by the acquisition of antibiotic resistance, we also reanalyzed transcriptomes of the Tet^R^ and WT strains with and without tetracycline treatment (32). In the absence of tetracycline, the Tet^R^ strain differentially expressed 197 genes (DEGs; adjusted *P* value < 0.05 and absolute log_2_ fold change >1) relative to the WT strain, including 65 metabolic genes, seven transcription factors (TFs) and four efflux pump (EP)-related genes (including the *acrAB* operon) (Fig. 1A). Functional enrichment on the dysregulated gene set revealed that 13 functional terms were significantly perturbed in the Tet^R^ strain (Fig. 1B; Table S2). Of note was differential regulation of 33 fermentation-related genes (randomized permutation test *P* value < 0.01), including the *frd* operon, *adhE*, *fumC*, and *ldhA* (which had *P* value < 0.05) (Fig. S1). Notably, upregulation of the *acrAB* operon and *acrZ* was consistent with known mechanisms of resistance to tetracycline and other antibiotics (Fig. S2A) (30, 37, 38). Disruption of the AcrAB efflux pump has been demonstrated to reduce the MIC of tetracycline to 0.5 μg/mL, which was 4-fold lower than for wild-type E. coli K-12 (reported as 2 μg/mL) (30), and also the overall fitness in the presence of tetracycline (39) (Fig. S2B).

10.1128/msystems.00904-22.1 FIG S1 Relative transcript level changes of fermentation-related genes in the Tet^R^ strain. The heat map shows log_2_ fold change in transcript levels of fermentation-related genes (compiled from the EcoCyc database) (102) in the wild-type (WT) and Tet^R^ strains with (+) and without (−) tetracycline treatment. The fold changes are relative to transcript levels in the WT strain without tetracycline treatment. Tetracycline concentrations used for WT and Tet^R^ strains were 0.25 and 16 μg/mL, respectively. Transcriptomics data were sourced from Händel et al. (32). Download FIG S1, PDF file, 0.04 MB. Copyright © 2022 Arrieta-Ortiz et al. 2022 Arrieta-Ortiz et al. https://creativecommons.org/licenses/by/4.0/ This content is distributed under the terms of the Creative Commons Attribution 4.0 International license.

10.1128/msystems.00904-22.2 FIG S2 The AcrAB efflux pump contributes to tetracycline resistance. (A) The *acrAB* operon and *acrZ* (that encodes a protein that interacts with the AcrAB pump and confers resistance to tetracycline) (37) were constitutively up-regulated in the Tet^R^ strain (32). Presence and absence of tetracycline are indicated as “(+)” and “(−)” next to the strain’s labels. Transcriptomics data were sourced from Händel et al. (32). (B) Fitness effect of single deletions of *acrA*, *acrB*, and *acrZ* during tetracycline treatment (with 0.25, 0.5, 0.75, and 1.0 μg/mL) in the E. coli K-12 strain (data sourced from Nichols et al. [39]). Fitness scores correspond to drug-gene interaction scores defined based on colony size of gene deletion strain library during drug treatment (39). The asterisk (*) symbol indicates that deletion of *acrA* and *acrB* were among the top 10 most deleterious on intrinsic tetracycline resistance phenotype across the library of ~3,800 gene knockouts. Boxes cover the 25^th^ to 75^th^ percentile ranges. The horizontal lines in boxes indicate median values. Download FIG S2, PDF file, 0.03 MB. Copyright © 2022 Arrieta-Ortiz et al. 2022 Arrieta-Ortiz et al. https://creativecommons.org/licenses/by/4.0/ This content is distributed under the terms of the Creative Commons Attribution 4.0 International license.

10.1128/msystems.00904-22.3 FIG S3 Hundreds of genes were identified as affecting Tet^R^ fitness during tetracycline treatment. (A) Heat map summarizing ALDEx2-based identification of gene deletions that significantly affect fitness (i.e., positive or negative effect based on over- and under-representation of the deletion mutants) on the WT and Tet^R^ backgrounds during competition assays of pooled single-gene deletion libraries in the presence of tetracycline (51). Only genes for which all detected knockout strains were statistically significant in the relevant comparison with respect to initial untreated time points were considered to have an impact on fitness. The asterisk symbol (*) indicates that there were 14 additional genes whose deletion was classified as deleterious in the WT strain but beneficial in the Tet^R^ background. Similarly, the double asterisk symbol (**) indicates that there were three additional genes whose deletion was classified as deleterious in the Tet^R^ strain but beneficial in the WT background. (B) General theme of term clusters identified by DAVID functional cluster enrichment analysis (91) in the Tet^R^-specific set of genes associated with beneficial and deleterious deletions. (C) Changes in interquartile log ratio transformed relative abundance profiles (with respect to [w. r. t.] initial time point [t0]) of transcription factor knockouts classified as contributing to WT and/or Tet^R^ fitness during tetracycline treatment according to ALDEx2 results. Download FIG S3, PDF file, 0.2 MB. Copyright © 2022 Arrieta-Ortiz et al. 2022 Arrieta-Ortiz et al. https://creativecommons.org/licenses/by/4.0/ This content is distributed under the terms of the Creative Commons Attribution 4.0 International license.

10.1128/msystems.00904-22.8 TABLE S2 Functional enrichment information of genes differentially expressed in the WT and Tet^R^ strains. Download Table S2, DOCX file, 0.02 MB. Copyright © 2022 Arrieta-Ortiz et al. 2022 Arrieta-Ortiz et al. https://creativecommons.org/licenses/by/4.0/ This content is distributed under the terms of the Creative Commons Attribution 4.0 International license.

The Tet^R^ strain differentially expressed nearly 10 times as many genes as the WT (with 896 versus 93 DEGs) in response to treatment with 0.25 and 16 μg/mL of tetracycline for WT and Tet^R^, respectively (Fig. 1A) (32). This differential regulation represented reprogramming of multiple processes (based on enrichment of 67 functional terms per the hypergeometric test), including the tricarboxylic acid (TCA) cycle (15 of 21 genes; *P* value = 1.7e-6), the electron transport chain (ETC, 13 of 23 genes; *P* value = 3.2e-4), and ATP synthase (four of eight genes; *P* value = 0.076) (Fig. 1B and C; Table S2). While tetracycline treatment did not result in substantial upregulation of the *acrAB* efflux pump in the Tet^R^ strain (i.e., variation in *acrAB* log_2_ fold change was less than 0.35; Fig. S2A), it induced the upregulation of at least four additional efflux pump genes (Fig. 1A). This suggested that the large-scale transcriptional remodeling, which was potentially mediated by 35 differentially expressed TFs (Fig. 1), might constitute a compensatory physiologic state that is triggered by increased efflux pump activity in the presence of tetracycline to ameliorate the loss of fitness associated with the resistance phenotype of the Tet^R^ strain (i.e., maximum growth rate and area under the growth curve of the Tet^R^ strain were ~30% lower than the corresponding fitness estimates of the WT strain; see below) (3). Specifically, repression of aerobic oxidative phosphorylation and induction of fermentation pathways suggested that a shift toward an anoxic physiologic state might be necessary to support the tetracycline resistance phenotype.

### A transcriptional program governed by 25 TFs underlies the physiological state required for tetracycline resistance.

**FIG 2:**
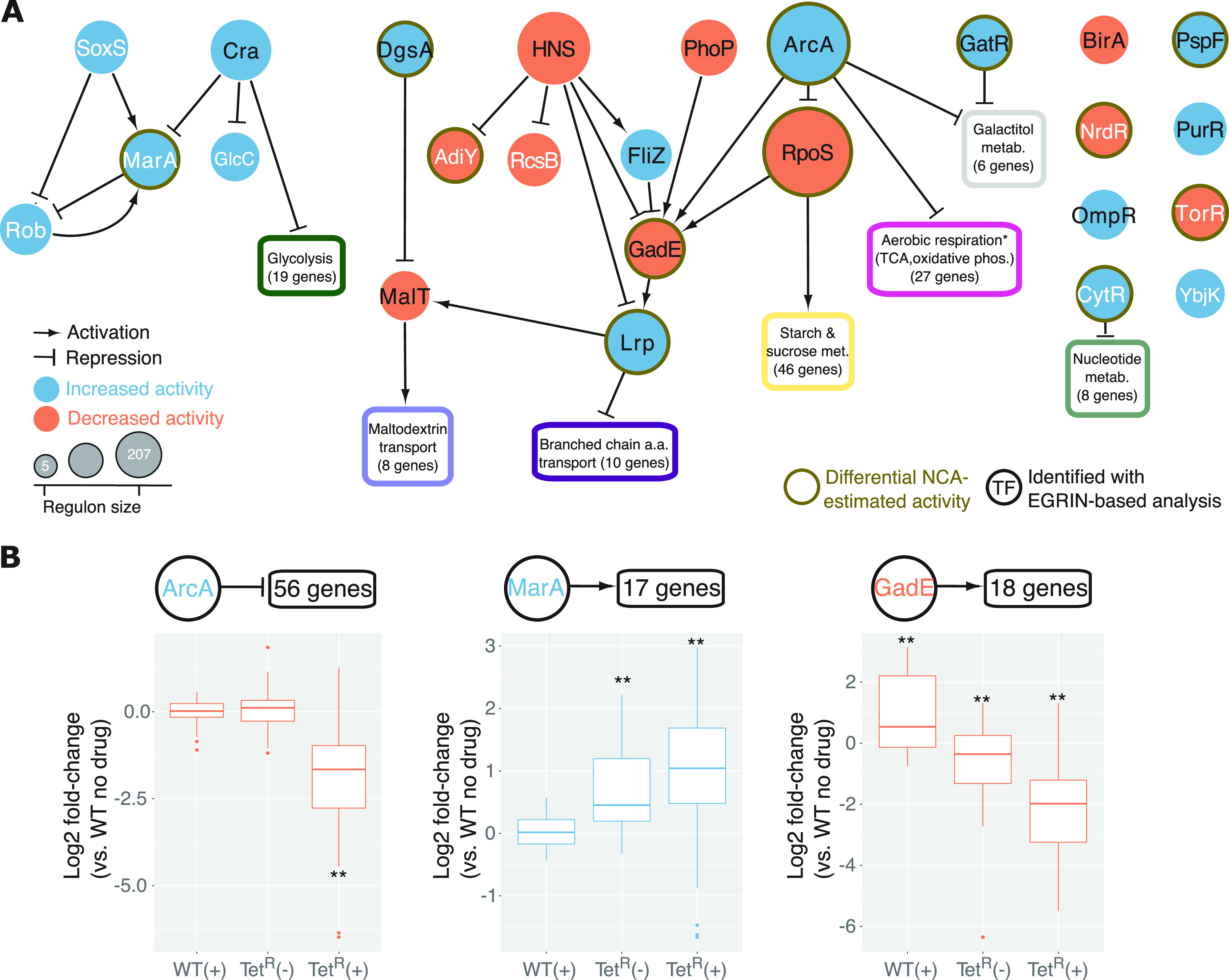
Regulatory circuits differentially active in tetracycline-resistant E. coli. (A) Subnetwork of 25 TFs (displayed as circles) implicated in driving transcriptional and metabolic reprogramming in the Tet^R^ strain (Table 1). TF autoregulation is not displayed. Functional enrichment (due to space constraints, only the functional term with the lowest *P* value is displayed) within subsets of six or more genes differentially expressed during Tet^R^ response to tetracycline and regulated by the same TF(s) implicated in Tet^R^ adaptive state (Table 1) are shown within boxes. The numbers in parentheses indicate the number of genes used to perform the functional enrichment analyses with DAVID (91) (not all genes may be associated with the shown functional term). The asterisk (*) indicates that the term was manually defined taking into account the overlap among multiple over-represented terms. TFs implicated by network component analysis (NCA) in regulatory and metabolic reprogramming of the Tet^R^ strain are indicated in nodes with a brown-colored border. Black font indicates TFs that were implicated based on significant overlap of their differentially regulated targets (in the Tet^R^ background) within coregulated gene modules in EGRIN (Environment and Gene Regulatory Influence Network) (44). The network was visualized using Cytoscape version 3.4.0 (105). (B) Fold change of the ArcA, MarA, and GadE regulons (transcriptional data from Händel et al. [32]) support their predicted increased (for ArcA and MarA) and decreased (for GadE) activity in mediating transcriptional reprogramming of the Tet^R^ strain at baseline and during adaptive response to tetracycline treatment. For dual regulators (i.e., activating and repressing different genes), the regulatory activity on the majority of their differentially expressed target genes (~80%) is shown. Boxplots display fold change of differentially expressed regulon members in the basal and/or adaptive states (number of regulated genes by each TF is indicated above each corresponding boxplot). Boxes cover the 25th and 75th percentile ranges. Horizontal lines in boxes indicate median values. Absence and presence of tetracycline treatment is indicated with “(−)” and “(+),” respectively. Statistical significance of the observed mean fold changes was evaluated by determining the null distribution of mean fold change in 10,000 random samplings of gene sets of similar size. *P* values are indicated with * (<0.05) and ** (<0.001).

We analyzed gene expression changes induced by gain of tetracycline resistance in the context of the transcriptional regulatory network to discover mechanisms responsible for regulatory and metabolic reprogramming of the Tet^R^ strain. We compiled a signed transcriptional regulatory network of E. coli based on curated positive or negative attributes to every TF-target gene interaction in RegulonDB (40). We then used the NetSurgeon algorithm (41) to identify within this transcriptional regulatory network the subset of TFs whose simulated overexpression and knockout explained the overall gene expression changes induced by gain of mutations (e.g., the *acrR* mutation) and treatment with tetracycline in the Tet^R^ strain. We hypothesized that the TF networks that were differentially active in the Tet^R^ strain upon tetracycline treatment were likely to be the mechanistic drivers of the compensatory physiologic state that was needed to support the resistance phenotype. Altogether, the NetSurgeon-based analysis implicated 25 TFs in mechanistically driving the differential regulation of 279 genes in the Tet^R^ strain, of which 209 genes were regulated by a subset of 15 TFs in response to tetracycline treatment (Table 1). Of the 25 TFs, 17 were part of two TF-TF network modules, suggesting coordination across their regulatory networks (Fig. 2A). While one subnetwork included TFs that were previously linked to AMR (MarA, SoxS, and Rob) (42), the other subnetwork was made up of TFs that control metabolic pathways (Lrp, MalT, GatR, and ArcA).

**TABLE 1:** TFs implicated in reprogramming transcriptional response of the Tet^R^ strain^*a*^

Transcription factor	Locus tag	Differential activity^*b*^	Response^*c*^	Regulon size^*d*^	Targets	
					Basal^*e*^	Adaptive^*f*^
RpoS	b2741	Decreased	Adaptive	207	20	69
ArcA	b4401	Increased	Adaptive	167	9	66
HNS	b1237	Decreased	Basal	146	18	33
Cra (FruR)	b0080	Increased	Adaptive	76	2	43
Lrp	b0889	Increased	Both	64	9	32
PhoP	b1130	Decreased	Basal	49	9	15
GadE	b3512	Decreased	Both	36	9	14
SoxS	b4062	Increased	Basal	33	7	13
MarA	b1531	Increased	Basal	33	11	11
RcsB	b2217	Decreased	Basal	33	7	10
PurR	b1658	Increased	Basal	31	10	10
Rob	b4396	Increased	Basal	22	8	7
FliZ	b1921	Increased	Basal	20	6	6
CytR	b3934	Increased	Adaptive	13	0	8
OmpR	b3405	Increased	Both	13	4	6
TorR	b0995	Decreased	Basal	12	3	4
MalT	b3418	Decreased	Adaptive	10	0	8
DgsA	b1594	Increased	Adaptive	10	0	7
NrdR	b0413	Decreased	Adaptive	9	1	4
AdiY	b4116	Decreased	Both	8	3	5
YbjK	b0846	Increased	Adaptive	8	1	5
PspF	b1303	Increased	Both	7	4	5
GatR	b4498	Increased	Adaptive	6	0	6
GlcC	b2980	Increased	Basal	6	3	3
BirA	b3973	Decreased	Adaptive	5	0	5

a DEG, differentially expressed gene; TF, transcription factor.

b “Increased” and “Decreased” indicate NetSurgeon-inferred change in the activity of each TF that explains the observed transcriptional response in a given treatment.

c “Basal” indicates the role of a TF in reprogramming transcriptional response of the Tet^R^ strain in the absence of tetracycline, whereas “Adaptive” indicates that the TF mediates transcriptional response of the Tet^R^ strain to tetracycline.

d Total number of genes directly regulated by each TF.

e Number of DEGs regulated by a TF in the absence of tetracycline.

f Number of DEGs regulated by a TF in the presence of tetracycline.

In a second independent approach, we used network component analysis to estimate the differential regulatory activity of TFs in Tet^R^ and WT with and without tetracycline treatment (43) (see Materials and Methods). The estimated regulatory activities of TFs were consistent with NetSurgeon predictions for 12 of the 25 TFs (indicated with brown node border in Fig. 2A). Finally, in a third approach, we discovered that 287 DEGs were statistically over-represented across 29 gene modules regulated by 15 (of the 25 TFs) within the previously developed Environment and Gene Regulatory Influence Network (EGRIN) model for E. coli (44). In summary, of the 25 TFs identified by NetSurgeon, 7 were also identified by the 2 orthogonal approaches. Altogether, the 15 TFs implicated in the response of Tet^R^ to tetracycline collectively regulated 23.3% (209 genes, hypergeometric test *P* value < 1e-26) of all DEGs, including 6 additional TFs, explaining how the response might have propagated to other genes in the genome. Notably, the predicted increased and decreased activity of TFs were consistent with the changes in expression profiles of their corresponding regulons across strains and treatments (Fig. 2B).

ArcA, a global transcriptional regulator that is typically induced under microaerobic conditions (45), was implicated by all three approaches as a mechanistic driver of the tetracycline response in the Tet^R^ strain. ArcA is a master regulator of one of the two TF-TF subnetworks, directly regulating 66 DEGs and influencing regulation by at least four downstream TFs (Fig. 2A; Table 1). There was significant overlap between DEGs in the Tet^R^ response to tetracycline and DEGs in an *arcA* deletion strain in anaerobic conditions (46) (hypergeometric test *P* value <1e-11). Importantly, ArcA is a known repressor of most genes of the TCA cycle and ETC (47) (Fig. 2A), and both processes were significantly downregulated in the Tet^R^ strain in the presence of tetracycline, which could have potentially perturbed NADH/NAD ratio and disrupted energy production via aerobic respiration. ArcA directly coordinates repression of the TCA cycle with activation of overflow metabolism (48), which is a fermentation mechanism to generate energy, albeit at lower efficiency, to cope with changes in demand for protein, energy, and biomass production under changing growth conditions (49, 50). Interestingly, fermentation genes were expressed at a higher level in the Tet^R^ strain even without tetracycline treatment, although the expression of TCA genes was downregulated only in the presence of tetracycline (Fig. 1C; Fig. S1). Based on these observations, we hypothesized that fitness loss associated with gain of efflux-mediated resistance to tetracycline in the Tet^R^ strain is compensated by an ArcA-mediated shift toward energy production by fermentation (48).

### Genome-wide CRISPR screen corroborates network-predicted mechanisms underlying a compensatory physiologic state that supports tetracycline resistance.

**FIG 3:**
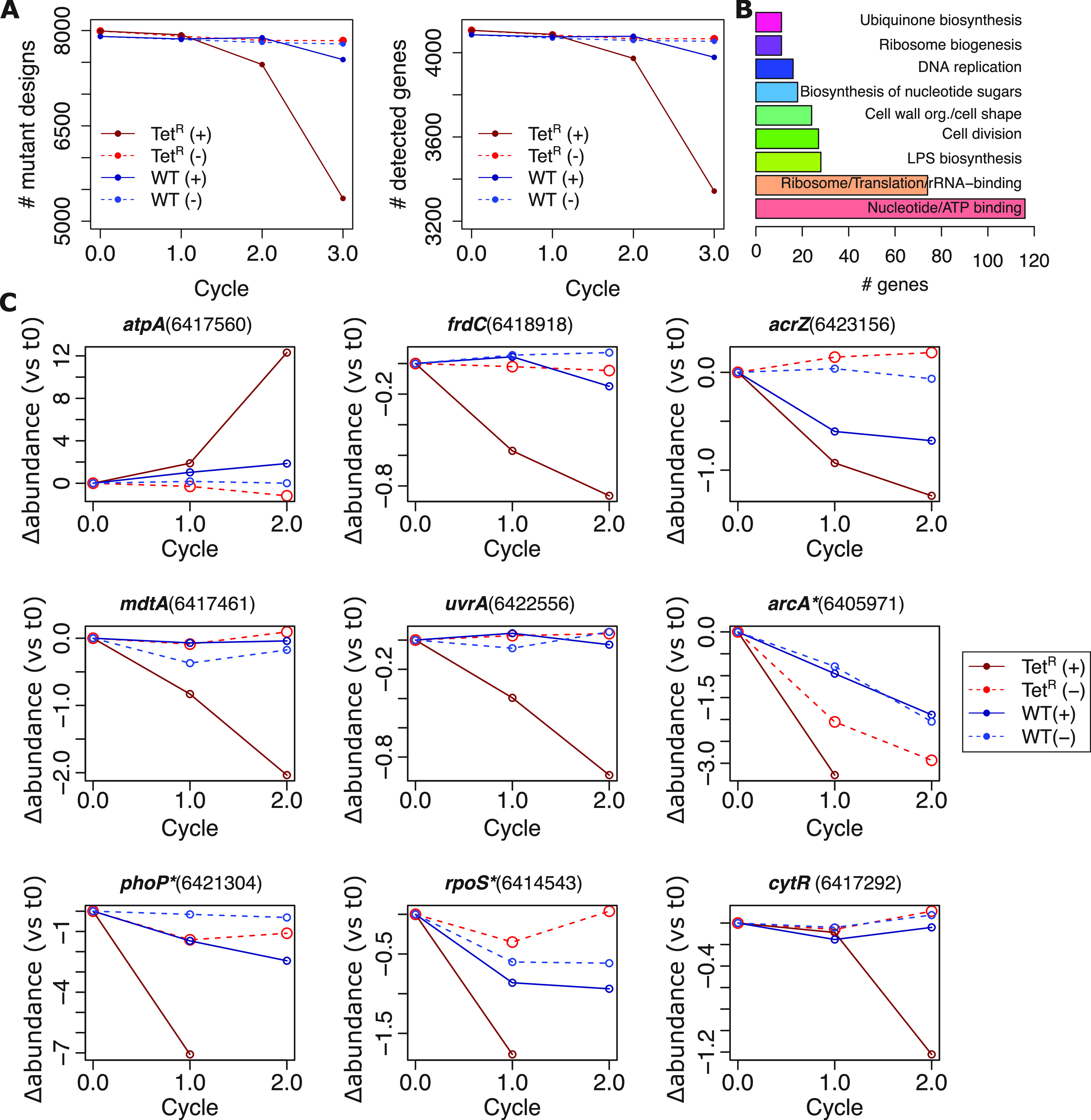
Gain of tetracycline resistance is associated with loss of robustness to genomic perturbations in the Tet^R^ strain. (A, left) Number of gene knockouts (KOs) detected during competitive growth assay in Tet^R^ and WT backgrounds with and without tetracycline treatment. Genome-wide KO libraries included on average two designs per gene, totaling 8,271 KOs. For a given time point, KOs that were not detected (i.e., <10 reads) in any of the four biological replicates were considered “dropouts.” (Right) Aggregation by gene. Genes were considered undetected if both KO designs were dropouts. (B) General theme of functional term clusters identified by DAVID functional annotation clustering (91) in the set of 726 genes depleted in Tet^R^ due to tetracycline treatment at the end of the competition assay (t3). (C) Changes in ALDEx2 (51) estimated interquartile log ratio (IQLR) transformed relative abundance (each cycle normalized to the initial time point, t0) of selected KOs during growth assays of Tet^R^ and WT genome-wide single-gene deletion libraries. For each selected gene, the profile of difference in abundance (i.e., the “diff.btw” scores computed by ALDEx2) of a single design KO is shown (design ID indicated in parenthesis). KOs that were undetectable at t2 (<10 reads in any replicate) are indicated with an asterisk (*) next to the gene name. Relative abundance was not estimated for undetected designs. The absence and presence of tetracycline in the experiments are indicated with the “(−)” and “(+)” symbols next to the strain background labels, respectively. LPS, lipopolysaccharide.

We investigated how each gene in the E. coli genome contributed to the compensatory physiologic state required to support the tetracycline resistance phenotype by performing an unbiased genome-wide CRISPR knockout (KO) screen. In brief, we constructed genome-wide knockout libraries using the Onyx Digital Genome Engineering Platform. The KO library consisted of 8,271 mutants, representing approximately two knockout designs for each gene in the Tet^R^ and WT strain backgrounds. The libraries were independently grown in quadruplicate over three rounds of sequential growth cycles (t0 to t3) in batch cultures with and without tetracycline (see Materials and Methods for details). To track the relative abundance of each KO strain in the population throughout the experiment, we performed barcode sequencing of the starting cultures, as well as at the end of each cycle. To account for the compositional nature of the data, we quantified changes in barcode abundance as the interquartile log ratio (IQLR) using ALDEx2 (51). This allowed us to compare the barcode abundance for each gene KO at each growth cycle relative to its starting abundance (t0) while controlling for changes in the overall library composition (Fig. 3A).

Comparing across strain backgrounds (Tet^R^ and WT) and treatments (+/− tetracycline), the ALDEx2 analysis revealed a multitude of context-dependent effects. In the presence of tetracycline, for example, KOs in 1,261 genes affected fitness in the Tet^R^ background compared to only 363 genes in the WT background (Data Set S1). Strikingly, we observed that KOs in 874 genes significantly improved fitness uniquely in the context of tetracycline treatment in the Tet^R^ strain background (Fig. S3A). Genes that were dispensable during tetracycline treatment in the Tet^R^ strain span a wide range of functions and processes, including iron homeostasis (17 genes), cell adhesion (21 genes), and aerobic metabolism (12 TCA-related genes and 7 ETC-related genes and *atpE*, which encodes a subunit of the ATP synthase) (Fig. S3B).

10.1128/msystems.00904-22.9 DATA SET S1 Read counts and output of ALDEx2-based analysis of sequencing data from competition assays of WT and Tet^R^ pooled genome-wide libraries of single-gene deletions. Download Data Set S1, XLSX file, 12.4 MB. Copyright © 2022 Arrieta-Ortiz et al. 2022 Arrieta-Ortiz et al. https://creativecommons.org/licenses/by/4.0/ This content is distributed under the terms of the Creative Commons Attribution 4.0 International license.

We also identified genes that became important for growth during tetracycline treatment, particularly in the Tet^R^ strain background. This finding was supported by two analyses: First, we observed many instances in which both KO designs for a gene were undetectable by the end of the experiment following treatment with tetracycline in the Tet^R^ background (“dropouts”; 82 genes in WT background compared to 726 genes in the Tet^R^ background [with 43 genes in common]) (Fig. 3A; Data Set S2). These 726 dropout genes included the AcrAB efflux pump-related genes *acrB* and *acrR*, both carrying SNPs in the Tet^R^ genome (Table S1), and the *arcA* and *arcB* genes, which encode the ArcB-ArcA two-component system (52). Translation-related genes encoding ribosomal subunits, ribosome biogenesis, and rRNA binding were also among the KOs not detected at the end of the experiment (Fig. 3B). Second, relative abundance analysis with ALDEx2, which accounted for potential compositional bias in strain abundance (see above), corroborated significant purifying selection of 308 single-gene KO strains during tetracycline treatment of the Tet^R^ strain. Within this group, 234 single-gene KO strains (including *arcA*) were under purifying selection only on the Tet^R^ strain (Fig. S3A). Gene sets identified by both analyses were significantly similar (hypergeometric test *P* value = 0.012). As expected, KOs identified with ALDEx2 as having a deleterious effect included genes associated with varied mechanisms of antimicrobial resistance, for example, *acrZ*, a component of the AcrAB efflux pump (37); *mdtA*, a member of a resistance-nodulation-division (RND) multidrug efflux pump (53); and *uvrA*, an excision repair system protein (Fig. 3C) (54, 55). Notably, a significant number of genes that contributed fitness to the Tet^R^ strain in the presence of tetracycline were associated with anaerobic respiration, and included menaquinone biosynthesis genes (*menA*, *menB*, *menC*, *menE*, and *menH*) (DAVID functional term adjusted *P* value = 5.8e-2) (Fig. S3B), fumarate reductase (*frdB* and *frdC*), anaerobic glycerol-3-phosphate dehydrogenase (*glpC*), selenate reductase (*ynfF*), formate dehydrogenase-N subunit (*fdnH*), hydrogenase 2 membrane subunit (*hybB*), and malate dehydrogenase (*mdh*) (56–63) (Fig. S4). Together with the transcriptome analysis, these results strongly suggest that a distinct anaerobic state supports tetracycline resistance in the Tet^R^ background.

10.1128/msystems.00904-22.4 FIG S4 Abundance profiles of anaerobic related gene knockouts during competition assays. Changes in ALDEx2 (51) estimated interquartile log ratio (IQLR) transformed relative abundance profiles (each cycle normalized to the initial time point [t0]) of *menB* (A), *frdB* (B), *hybB* (C), and *mdh* (D) gene deletion mutants during competitive assays of Tet^R^ and WT genome-wide single-gene deletion libraries. For each gene, the profile of difference in abundance (i.e., the “diff.btw” scores computed by ALDEx2) of a single knockout (design ID indicated in parentheses) is shown. For each knockout design, the displayed values correspond to the median difference in ALDEx2-estimated IQLR values. Absence and presence of tetracycline in the experiments are indicated with (−) and (+) symbols next to the strain labels, respectively. Download FIG S4, PDF file, 0.07 MB. Copyright © 2022 Arrieta-Ortiz et al. 2022 Arrieta-Ortiz et al. https://creativecommons.org/licenses/by/4.0/ This content is distributed under the terms of the Creative Commons Attribution 4.0 International license.

10.1128/msystems.00904-22.10 DATA SET S2 List of undetected KO designs and gene KOs at different time points of the growth competition assays of libraries of single-gene deletions. Download Data Set S2, XLSX file, 0.05 MB. Copyright © 2022 Arrieta-Ortiz et al. 2022 Arrieta-Ortiz et al. https://creativecommons.org/licenses/by/4.0/ This content is distributed under the terms of the Creative Commons Attribution 4.0 International license.

To understand whether transcriptional regulatory mechanisms drive large-scale physiological remodeling in the Tet^R^ background, we analyzed the fitness effects of TFs in the high-throughput CRISPR KO screen. Fifteen TF KOs significantly reduced fitness in the Tet^R^ background, but, interestingly, only following tetracycline treatment (Fig. S3C). This finding underscores the importance of global transcriptional reprogramming for the tetracycline resistance phenotype in the Tet^R^ background (Fig. 1 and 2A). Reassuringly, there was a high degree of overlap between the CRISPR screen in the presence of tetracycline and our network-based approach (10 of 24 TFs identified by the network-based approach and with KO designs, including *arcA*, *cytR*, *phoP*, and *rpoS*) (Fig. 2A and 3C). Notably, *arcA* and *phoP* were among the most deleterious KOs in the CRISPR screen after the first growth cycle. Together, these findings suggest that the tetracycline resistance phenotype in Tet^R^ E. coli is associated with transcriptional coordination of fermentative carbon metabolism by ArcA, acid stress response by PhoP (64), generalized stress response by RpoS (65), and carbon and nucleotide metabolism by CytR (66, 67) (Fig. 3C). These findings also implicate ArcA as a key TF that drives the physiological shift of the Tet^R^ strain to a fermentative state following treatment with tetracycline.

### ArcA activity ameliorates the fitness cost of tetracycline resistance.

**FIG 4:**
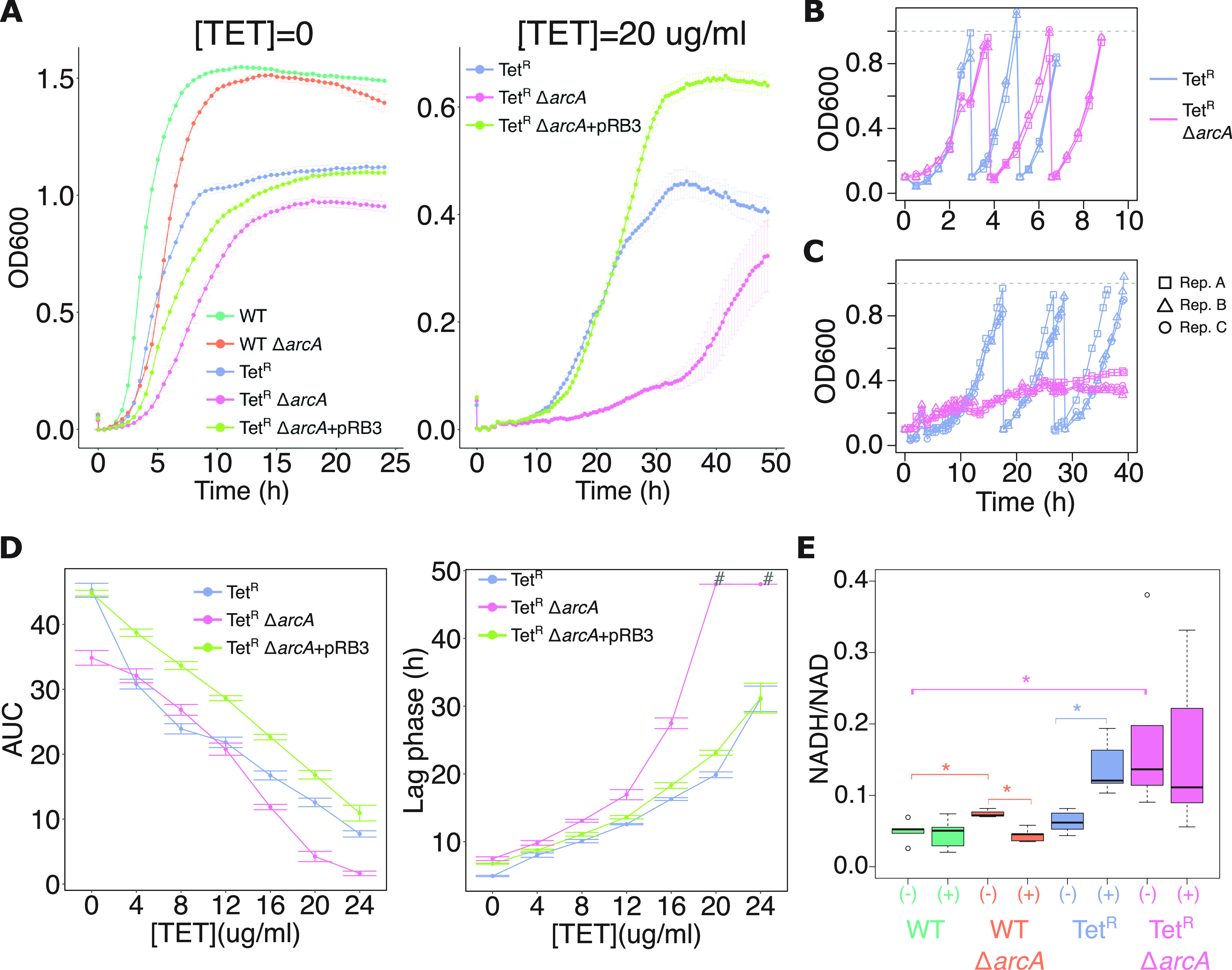
ArcA restores fitness of tetracycline-resistant E. coli. (A) Representative growth curves in LB medium for 48h with and without tetracycline (TET). Six replicates (Rep.) (i.e., three biological replicates with two replicates each) per strain were used. Points indicate average values and error bars indicate standard deviation. Effect of *arcA* deletion on Tet^R^ fitness in LB broth cultures without (B) or with 20 μg/mL of tetracycline (C) over three growth cycles is shown. In each cycle, cultures were started at an optical density at 600 nm (OD_600_) of 0.1 and grown to an OD_600_ of 1.0 (indicated by the gray dashed lines) and thereafter diluted in fresh medium to OD_600_ of 0.1 to reinitiate a new cycle of growth. (D) Area under the growth curve (AUC) and lag phase (approximated by Growthcurver-estimated time of inflection, the time required to achieve half of maximal OD_600_ [68]) for seven different concentrations of tetracycline. The # symbol indicates samples in which Growthcurver-estimated time of inflection was longer than the actual duration of the experiment and therefore adjusted to 48 h. (E) NADH/NAD ratio during log phase of the WT, WT Δ*arcA*, Tet^R^, and Tet^R^ Δ*arcA* strains in the absence (indicated with “(−)”) and presence (indicated with “(+)”) of tetracycline. NADH/NAD ratios in the absence of tetracycline were compared with respect to untreated WT using a Welch’s *t* test. Similarly, ratios in the tetracycline-treated condition versus the untreated condition were compared for each strain; *P* values < 0.05 are indicated with the asterisk (*) symbol. Tet^R^ Δ*arcA*+pRB3, *arcA* deletion strain complemented with episomal copy of *arcA*.

We further investigated the importance of ArcA activity for tetracycline resistance by constructing an in-frame knockout (Δ*arcA*) in the WT and Tet^R^ strain backgrounds and quantifying the overall fitness of both sets of parental and Δ*arcA* strains by calculating the area under the growth curve in batch cultures (68–70) (Fig. 4A to D). The fitness analysis demonstrated that gain of tetracycline resistance had significantly increased the relative importance of ArcA in the Tet^R^ strain (Fig. 4A), especially in the presence of high doses of tetracycline (16 to 24 μg/mL). In fact, in the presence of tetracycline, the Tet^R^ Δ*arcA* strain was unable to achieve half the overall carrying capacity of the parental strain even after extended culturing (Fig. 4B and C). In stark contrast, the *arcA* deletion had a more subtle effect in the parental WT strain (Fig. S5). The fitness defect of the Tet^R^ Δ*arcA* strain during tetracycline treatment was completely reversed upon complementation with an episomal copy of *arcA* (Fig. 4D).

10.1128/msystems.00904-22.5 FIG S5 Effect of *arcA* deletion on WT fitness in LB broth cultures. Cultures without (A) or with (B) 0.75 μg/mL of tetracycline over three growth cycles are shown. In each cycle, cultures were started at an optical density at 600 nm (OD_600_) of 0.1, grown to an OD_600_ of 1. 0 (indicated by the gray dashed lines), and thereafter diluted in fresh medium to OD_600_ of 0.1 to reinitiate a new cycle of growth. Rep, Replicates. Download FIG S5, PDF file, 0.03 MB. Copyright © 2022 Arrieta-Ortiz et al. 2022 Arrieta-Ortiz et al. https://creativecommons.org/licenses/by/4.0/ This content is distributed under the terms of the Creative Commons Attribution 4.0 International license.

It has been demonstrated that increase in intracellular NADH/NAD ratio, such as during fast growth (49), triggers ArcA (71, 72), which then mediates repression of the TCA cycle and activation of overflow metabolism to prevent further redox imbalance (49). Consistent with this sequence of events, we observed that tetracycline treatment significantly increased the intracellular NADH/NAD ratio in the Tet^R^ strain, which explains the increased activity of ArcA with gain of tetracycline resistance (Fig. 4E). As expected, deletion of *arcA* resulted in constitutive dysregulation of NADH/NAD ratio irrespective of tetracycline treatment, presumably due to disruption of the ArcA-mediated feedback mechanism to manage redox balance (Fig. 4E). We propose based on these results that ArcA plays a central role in modulating redox balance to support increased efflux-mediated tetracycline resistance phenotype.

### Molecules that mimic ArcA knockout phenotype disrupt efflux-mediated tetracycline resistance.

**FIG 5:**
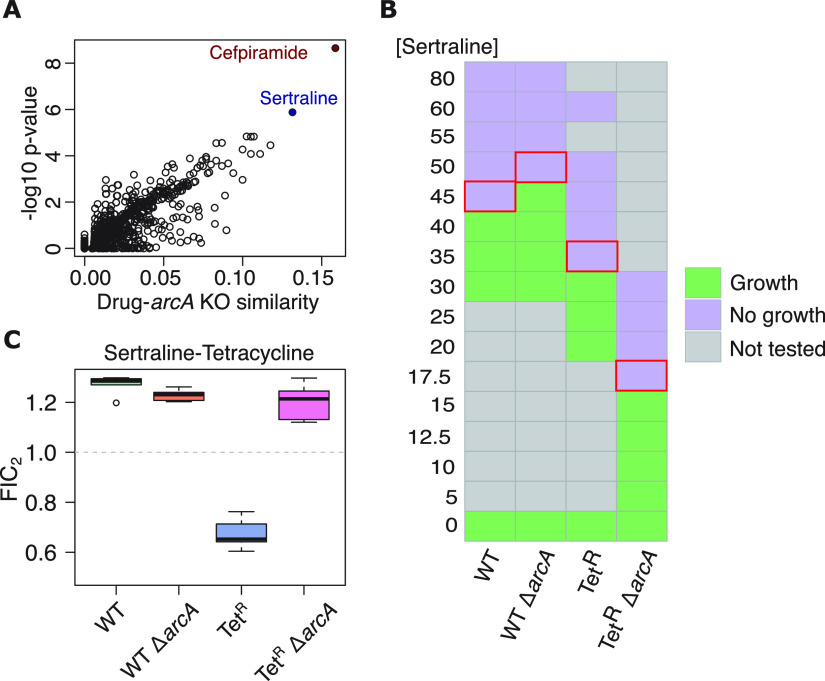
ArcA plays a central role in the synergistic effect of the sertraline-tetracycline combination in the tetracycline-resistant E. coli. (A) Analysis of similarity (estimated by pairwise comparison of *Z*-score-normalized profiles) between metabolic profiles of E. coli treated with 1,279 Food and Drug Administration (FDA)-approved compounds (25) and the metabolic profile of the *arcA* E. coli deletion strain (74), computed by Campos and Zampieri (25), identified sertraline and cefpiramide as promising candidate ArcA inhibitors. (B) Growth assays in a microdilution series (0 to 80 μg/mL) were performed to assess dose response and susceptibility of different strains to sertraline (three biological replicates each one with three replicates for a total of nine replicates per strain/concentration). “Growth” indicates that for at least two replicates, the increase in OD_600_ was higher than two times the maximum increase in OD_600_ of control wells (inoculated with LB medium) after 16-h incubation. Red rectangles indicate the lowest concentration at which growth was not observed. (C) Results of DiaMOND assays to estimate fractional inhibitory concentration (FIC_2_) for the sertraline-tetracycline combinations in the four strains (76). The data for five biological replicates per strain (six for Tet^R^) are shown.

In order to identify drugs that would simulate an *arcA* knockout phenotype, we leveraged available comparisons of metabolome profiles of drug-treated and single-gene deletion strains of E. coli (25). Among the 1,279 Food and Drug Administration (FDA)-approved compounds in this analysis, two compounds, sertraline (a serotonin reuptake inhibitor used as antidepressant) (73) and cefpiramide (a third-generation cephalosporin), generated metabolome profiles that were most similar to the metabolome of the *arcA* deletion strain (74) (Fig. 5A). Reciprocally, of ~3,800 gene deletions, *arcA* deletion was among the top 20 strains whose metabolomes were most similar to metabolome profiles generated by the two compounds. We reasoned that for the metabolome similarities to be physiologically meaningful and clinically relevant, the concentration of the drug needed to be equal to or less than the MIC for the Tet^R^ strain. While the sertraline concentration used in the metabolomics study was within MIC for Tet^R^, the cefpiramide concentration in the metabolic profiling study (100 μM, 61.3 μg/mL) (25) was higher than the estimated MIC (<20 μg/mL). Nonetheless, we performed a DiaMOND assay to evaluate a potential interaction between cefpiramide and tetracycline. An additive interaction was detected in the Tet^R^ strain (fractional inhibitory concentration [FIC_2_] score of ~1.2). Upon further exploration, we discovered that the metabolic pathways targeted by cefpiramide treatment were different from those affected upon the deletion of *arcA* (Fig. S6). Hence, this analysis demonstrated that while the metabolome similarity analysis helps to shortlist compounds, further analysis at the metabolic pathway level may be needed to ascertain whether the compounds might be synergistic due to a double hit on the same pathways (Fig. S6). We excluded cefpiramide from further analysis and proceeded to test whether sertraline could resensitize Tet^R^ E. coli to tetracycline.

10.1128/msystems.00904-22.6 FIG S6 E. coli metabolic pathways perturbed by sertraline treatment, cefpiramide treatment, and *arcA* deletion. The *Z*-score normalized metabolic profiles of E. coli treated with sertraline and cefpiramide (reported by Campos and Zampieri) (25), and the *Z* score normalized metabolic profile of the E. coli *arcA* deletion strain (reported by Fuhrer et al. [74]) were analyzed using MetaboAnalyst 5.0 (94), as explained under Materials and Methods. Only metabolic pathways over-represented (with false discovery rate [FDR] hypergeometric test adjusted *P* values ≤ 0.25) in at least one of the three conditions are shown. Pathways associated with both sertraline treatment and *arcA* deletion are shown in red bold font. Download FIG S6, PDF file, 0.2 MB. Copyright © 2022 Arrieta-Ortiz et al. 2022 Arrieta-Ortiz et al. https://creativecommons.org/licenses/by/4.0/ This content is distributed under the terms of the Creative Commons Attribution 4.0 International license.

Next, we also analyzed metabolomes of E. coli upon treatment with sertraline and deletion of *arcA* to discover that each perturbation individually resulted in the overactivation of TCA cycle, pyruvate metabolism, pentose phosphate pathway, butanoate metabolism, and inositol phosphate metabolism (Fig. S6). These findings suggested that treatment of the Tet^R^ Δ*arcA* strain with sertraline would likely lead to accumulation of toxic reactive oxygen species (ROS) due to overactivation of the TCA cycle (75). Consistent with the increased importance of ArcA-induced response in tetracycline resistance, sertraline (previously reported as bactericidal against E. coli [73]) was significantly more potent on the Tet^R^ strain (MIC: 35 μg/mL), relative to the WT strain (MIC, 45 μg/mL). Notably, deletion of *arcA* slightly increased the MIC of sertraline on WT (MIC, 50 μg/mL). In contrast, sertraline was twice as potent upon knocking out *arcA* in the Tet^R^ background (MIC 17.5 μg/mL; Fig. 5B). In other words, sertraline may kill E. coli by disrupting the ArcA network, and its increased activity on the Tet^R^ Δ*arcA* strain might amount to a double hit on the same network, providing a mechanistic explanation for why the two drugs are synergistic on Tet^R^ E. coli (73).

Finally, we performed a DiaMOND assay (76) to experimentally test the potential mechanism of synergy between tetracycline and sertraline by investigating dose-dependent combinatorial effects of the two drugs on WT and Tet^R^ strains with and without *arcA* deletion (Fig. 5C). The activity of the tetracycline-sertraline combination was additive on the WT strain (FIC_2_ score, ~1.27), with minimal change upon deletion of *arcA* (FIC_2_ score, ~1.23). In stark contrast, the drug combination was potentially synergistic on the Tet^R^ strain (FIC_2_ score, ~0.67), in agreement with previous reports of synergy between sertraline and tetracycline (73). Remarkably, the drug combination was additive on the Tet^R^ Δ*arcA* strain (FIC_2_ score, 1.2), demonstrating unequivocally that the suggested synergy between tetracycline and sertraline emerges from disruption of the compensatory physiologic state that is mechanistically generated by increased ArcA activity.

## DISCUSSION

**FIG 6:**
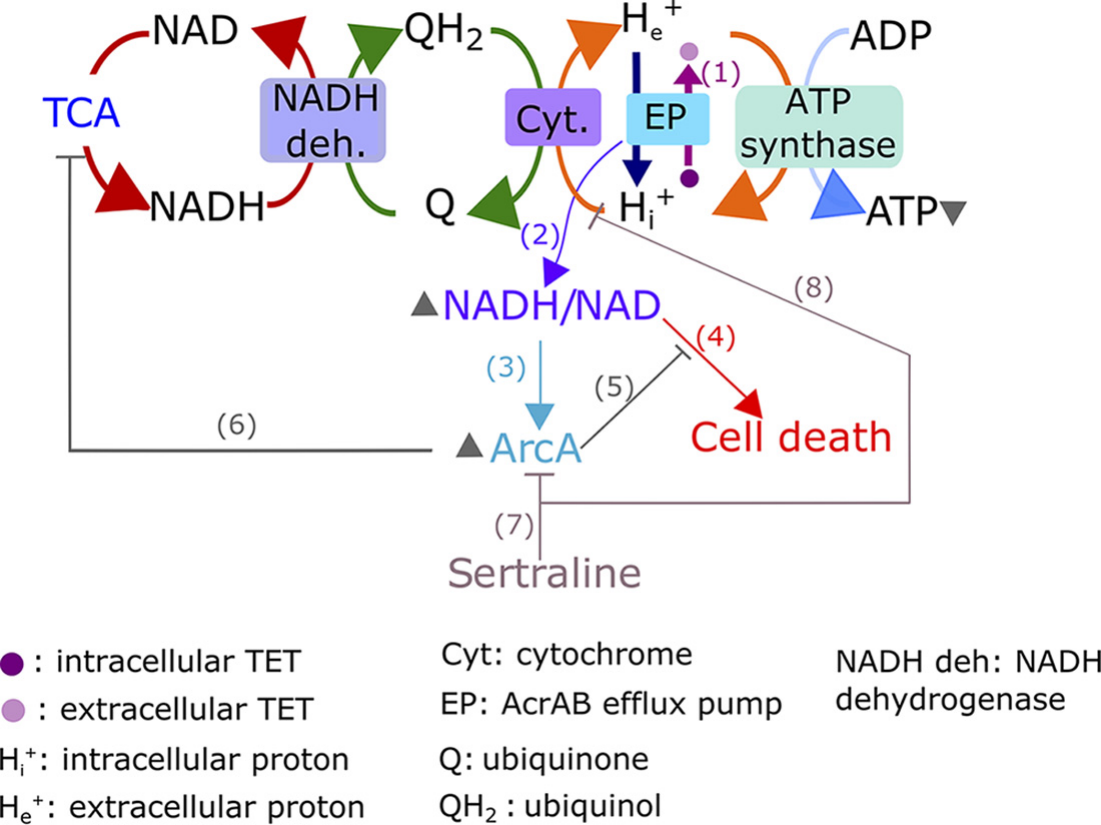
Summary overview of the ArcA-driven compensatory mechanism for tetracycline resistance and its connection with efflux pump mediated resistance. In the absence of tetracycline, Tet^R^ upregulates the *acrAB* efflux pump (EP), which in turn causes upregulation of fermentation-related genes (Fig. S1 and S2). The activity of AcrAB increases in the presence of tetracycline (30) (edge 1), driving dramatic changes in metabolism. We hypothesize that AcrAB disrupts the functions of other transporters by crowding the membrane, consuming the proton motive force (PMF), causing an increase in NADH/NAD ratio (edge 2) (Fig. 4E), which activates ArcA (71) (edge 3) (Fig. 2A). The potential toxic consequence of higher NADH/NAD ratio (49) (edge 4) is alleviated by ArcA (edge 5) through downregulation of the tricarboxylic acid (TCA) cycle (47) (edge 6). Finally, sertraline represses the ArcA network (edge 7) (Fig. 5A; Fig. S6) and putatively inhibits the PMF (73) (edge 8) to synergistically potentiate the bacteriostatic effect of tetracycline. Relative increases in abundance or activity are indicated with an upward pointing arrowhead, next to the corresponding molecule. Decreased concentration is indicated with a downward pointing arrowhead.

We have discovered that global remodeling of transcription by a network of at least 25 TFs generates a novel metabolic state to compensate for loss of fitness that accompanies the gain of tetracycline resistance in E. coli. Interestingly, while the resistance mutations resulted in constitutive overexpression of the *acrAB* operon in the Tet^R^ strain, the global transcriptional remodeling manifested in a dramatic manner only during tetracycline treatment, suggesting that it was a downstream consequence of the increased activity of the AcrAB efflux pump. We propose a model to explain how increased efflux triggers a compensatory physiologic state to support tetracycline resistance in Tet^R^ E. coli (Fig. 6). AcrAB is an efflux pump of the RND superfamily that consumes the proton motive force (PMF) to expel intracellular substrates, tetracycline in this case (53). Hence, AcrAB competes with the ETC and ATP synthase both for space in the membrane and for the PMF, which is required for ATP synthesis (49). This competition may reduce oxidation of NADH molecules by the ETC, and the resulting increase in NADH/NAD ratio triggers ArcA. ArcA acts by repressing the TCA cycle and redirecting metabolic flux away from oxidative phosphorylation and toward overflow metabolism, which serves as an alternative source of energy (48, 50, 71, 72). This hypothesis is supported by two previously reported observations: (i) deleting either of the two repressors (*marR* and *acrR*) of the *acrAB* operon resulted in the increased secretion of acetate, a by-product of overflow metabolism (77); and (ii) mutations in *acrB* significantly reduced the rate of oxygen uptake (77). The downregulation of the TCA cycle may also serve to mitigate oxidative stress by preventing the production of ROS (75, 78). The global remodeling of the respiration and energy production pathways appears to be a generalized mechanism for restoring fitness across AMR pathogens, including P. aeruginosa (19) and Chromobacterium violaceum (24), that also manifest high intracellular NADH levels upon gaining resistance to diverse antibiotics through the increased expression of RND efflux pumps.

Amplification of the fitness cost of tetracycline resistance upon disrupting the master regulator (ArcA) of the compensatory metabolic state demonstrated how a network-based approach can rationally identify new vulnerabilities that emerge as a consequence of gaining resistance, because knocking out ArcA had a minor fitness consequence in the WT background (Fig. 3C and 4A). Having identified ArcA as a new vulnerability in the Tet^R^ strain, we were able to identify secondary molecule(s) that could target the compensatory physiologic state by leveraging the similarities between global metabolome changes in E. coli across a library of single-gene deletion strains and a drug library screen (25). By mining this publicly available metabolome comparison, we rank-prioritized the most likely drugs in the screen that could disrupt the ArcA network to potentiate tetracycline action. Sertraline, which was among the top ranking candidates, was proposed to potentiate tetracycline action by blocking the PMF and indirectly inhibiting its efflux (73, 79, 80), albeit by a different plasmid-encoded TetA pump (27, 73). The loss of synergistic action of tetracycline-sertraline combination upon deleting *arcA* demonstrated the mechanism of synergy (Fig. 5C) but also revealed how Tet^R^ E. coli could (re)gain resistance and tolerance to this drug combination through a single regulatory mutation or transcriptional reprogramming of a single TF. Notably, the network analysis in this study implicated at least 25 TFs and their networks as mechanistic drivers of the compensatory physiologic state required to support the tetracycline resistance phenotype of Tet^R^. This finding was supported by the dramatically different landscape of genome-wide fitness in the Tet^R^ strain background compared to the WT, which also illustrated how gaining resistance, at least by an efflux mechanism, is associated with system-wide trade-offs across multiple processes (Fig. 3). The complexity of this regulatory and metabolic network reprogramming also suggests that there are multiple routes through which a pathogen like E. coli could escape antibiotic treatment to gain resistance, explaining why we need multidrug combinations to combat antibiotic tolerance and resistance (81–83).

Formulating a multidrug regimen is particularly challenging because the numbers of combinations that need to be tested is too large, even for a high-throughput drug screen (84). A network-based approach, like the one described in this study and previously (81), will prove valuable in this effort because it uses a mechanistic model of a gene regulatory network underlying tolerance or resistance phenotypes to rank-prioritize combinations of molecules that target multiple vulnerabilities within a pathogen. We have demonstrated that this strategy could enable the recovery of “lost” antibiotics by identifying new vulnerabilities that emerge within transcriptional and metabolic networks to manage trade-off in fitness upon gain of resistance (3, 19). This approach can be combined with laboratory evolution experiments to delineate trajectories of antibiotic resistance (85) and design drug combinations to preemptively curtail the emergence and spread of AMR. For instance, antibiotic-tolerant strains of E. coli gained resistance at a higher rate than the wild-type strain, suggesting that drugs that target the tolerance networks within these strains could potentially delay or block the emergence of resistance (85). Another high-throughput screen of a library of TF deletion strains demonstrated that deletion of *arcA* suppresses gain of resistance to cefixime, ciprofloxacin, and chloramphenicol (78). We posit that a multidrug regimen formulated based on vulnerabilities within networks governed by ArcA and other TFs identified in this study could have generalized value in extending the life spans of a broad range of existing antibiotics and supplement the development of new antimicrobial compounds (5).

## MATERIALS AND METHODS

### E. coli strains and culturing conditions.

E. coli MG1655 (WT) and a lab-evolved tetracycline-resistant E. coli derived from the susceptible WT (Tet^R^) were kindly provided by Benno ter Kuile (32). *arcA* mutants were generated using the Red/ET recombination kit (Gene Bridges, Germany), following the manufacturer’s instructions. Using this approach, a kanamycin cassette was inserted between the 5′ and 3′ regions of *arcA*. *arcA* disruption was confirmed with PCR and Sanger sequencing. *arcA* deletion was complemented with the pRB3-*arcA* plasmid (86) kindly provided by Sangwei Lu. E. coli strains were grown on Luria-Bertani (LB) broth on agar and broth (with constant shaking) at 37°C and aerobic conditions.

### Assessment of *arcA* deletion effect on fitness.

Microdilution growth curves were performed in LB broth using a Bioscreen C instrument (Growth Curves USA, Piscataway, NJ). First, frozen cells were used to streak individual colonies in LB plates (without antibiotic for WT and WT Δ*arcA*, and with 4 μg/mL of tetracycline [Sigma-Aldrich] for Tet^R^, Tet^R^ Δ*arcA*, and Tet^R^ *arcA*^+^ [episomal complemented strain]). Isolated colonies were used to start tetracycline-free overnight cultures used as inoculum for the growth assays. Overnight cultures were adjusted to 0.01 Optical density at 600 nm (OD_600_), and 200-μL cultures with different concentrations of tetracycline (and other used compounds) were run at 37°C with continuous shaking. OD_600_ was measured every 30 min. Wells inoculated only with LB were included to measure background OD_600_ of the medium and as sterility controls. The Growthcurver R package (68) was used to fit a logistic equation to the OD_600_ data for each well after subtracting its minimum OD_600_ reading. Bacterial fitness was estimated in terms of the area under the growth curve (AUC) empirically determined by Growthcurver (68). The AUC value integrates multiple properties of the growth curve (69, 70).

For broth macrodilution experiments, four isolated colonies (from antibiotic-free LB plates for WT and WT Δ*arcA* and LB plates with 4 μg/mL of tetracycline for Tet^R^ and Tet^R^ Δ*arcA*) were used to start overnight LB cultures. Three overnight LB cultures per strain were diluted to 0.1 OD_600_ in a final volume of 10 mL (in disposable culture tubes; Fisherbrand) with tetracycline (0.75 μg/mL for WT and WT Δ*arcA* and 19.9 μg/mL for Tet^R^ and Tet^R^ Δ*arcA*) and without tetracycline (i.e., with addition of ethanol, used as solvent for tetracycline). As a control, the same volume of ethanol used for the tetracycline-treated samples was added to antibiotic-free cultures. These cultures were the starting point for the experiment. OD_600_ was periodically measured using a SPECTRONIC 200E spectrophotometer (Thermo Scientific). To maintain cultures in mid-log phase, the cultures were diluted back to 0.1 OD_600_ when they achieved an OD_600_ of 1.0. Due to practical constraints, the cultures were considered in the target OD_600_ range when they were in the 0.8 to 1.2 OD_600_ interval. Two sterile controls (containing only LB broth) were maintained throughout the experiments. All cultures were transferred after achieving the target OD_600_ range for a total of three growth cycles (i.e., from 0.1 OD_600_ to ~1.0 OD_600_). The two controls were also transferred and followed for a total of three cycles until the end of the experiment. Sub-MIC tetracycline concentrations were selected based on moderate inhibition effects in Growthcurver-estimated maximum growth rates of microdilution growth curves (~60% for WT and between 53% and 76% for Tet^R^ treated with 0.75 and 20 μg/mL of tetracycline, respectively), as performed previously by others (87).

### Sertraline inhibitory concentration determination.

We performed Bioscreen growth assays to assess the susceptibility of the different strains to sertraline (sertraline hydrochloride; Sigma-Aldrich). Growth assays covered a wide range of concentrations (for each compound/strain, we initially performed an exploratory experiment to define an optimal range including nine concentrations) for each compound of interest using three biological replicates (each one with three replicates for a total of nine replicates) per strain/concentration. Growth was defined as the instances in which two or more replicates increased their OD_600_ more than twice the maximum OD_600_ increment of the control wells (inoculated with sterile LB medium) after 16 h of incubation.

### DiaMOND assay to evaluate synergy of two-drug combinations.

We used the diagonal measurement of n-way drug interactions (DiaMOND) assay (76) (as described by Cokol-Cakmak et al. [88]) to evaluate the predicted ArcA-mediated synergy between tetracycline and selected compounds. Briefly, we first determined the single drug concentrations that reduced OD_600_ (measured after 16 h of growth) by half with respect to the drug-free condition (corresponding to the drug IC_50_), using a BioTek Epoch 2 instrument (BioTek, USA). Spline fitting and the R Stats package were used to interpolate the IC_50_ values in the analyzed dose-response curves. Then, the IC_50_ values of the two-drug combinations (between tetracycline and selected compounds in a 1:1 volume using the IC_50_ concentrations defined in the previous step) were determined. The fractional inhibitory concentration for the two-drug combinations (FIC_2_) under the Loewe additivity model was defined (76). Culture inoculums were prepared as described before. As the last step of the DiaMOND assay, five biological replicates (from independent colonies) were used to start five overnight cultures to measure the effect of the tetracycline-sertraline interaction. The raw data were visually inspected, and replicates with more than one potential IC_50_ or that grew better than expected (i.e., higher OD_600_) in concentrations above the estimated IC_50_ were removed due to low confidence on the IC_50_ estimation.

### NADH/NAD ratio measurements.

NADH and NAD ratios were measured using the Enzychrom NAD/NADH assay kit (Bioassay Systems), following the manufacturer’s instructions (and adding a sonication step of 20 s [89], before heating at 60°C for 5 min to lyse the bacterial cells). The equivalent of 1 mL of a 1.0 OD_600_ bacterial culture was used to measure NAD and NADH concentrations. To simultaneously capture all bacterial cultures in log phase, overnight LB cultures (started from isolated colonies as previously explained for growth experiments) were adjusted to slightly different OD_600_ values to correct for differences in fitness among strains. Specifically, in the absence of tetracycline, the initial OD_600_ values for WT, WT Δ*arcA*, Tet^R^, and Tet^R^ Δ*arcA* were 0.015, 0.025, 0.04, and 0.07, respectively. In the presence of tetracycline (0.75 μg/mL for WT and WT Δ*arcA* and 4 μg/mL for Tet^R^ and Tet^R^Δ*arcA*), the initial OD_600_ values were 0.02 (WT), 0.04 (WT Δ*arcA*), and 0.05 (Tet^R^ and Tet^R^Δ*arcA*). A tetracycline concentration of 0.75 μg/mL for WT and WT Δ*arcA* was selected based on its inhibitory effect (see above). For Tet^R^ and Tet^R^Δ*arcA*, a tetracycline concentration of 4 μg/mL was chosen based on the observation that fitness of both Tet^R^ and Tet^R^Δ*arcA* was similar at the selected concentration (Fig. 4D). The selection was intended to minimize the impact of fitness differences (already detectable in the antibiotic-free condition; Fig. 4A) in the measured NADH/NAD ratio. NADH and NAD concentrations were measured after ~2.25 h (for cultures without tetracycline) and ~5.25 h (for cultures with tetracycline) of growth at 37°C. Final results included three to six replicates per strain.

### Differential expression analysis of E. coli MG1655 and tetracycline-resistant MG1655 microarray data.

To characterize the adaptation of the Tet^R^ strain to tetracycline, we analyzed publicly available normalized microarray data for the WT and Tet^R^ strains in the presence and absence of tetracycline (Gene Expression Omnibus accession number GSE57084) reported by Händel et al. (32). Differential expression analysis of microarray data was performed using a Bayesian *t* test with the Cyber-T tool (90). Genes with adjusted *P* values < 0.05 and absolute log_2_ fold change >1 were considered differentially expressed. Transcriptional profiles of *arcA* deletion strains (GEO accession numbers GSE1121 and GSE46415) (46, 47) were analyzed using the same thresholds.

### Functional enrichment analysis.

Enrichment analyses of significantly up- and downregulated set of genes were independently performed using DAVID (91). Only functional terms with adjusted *P* values (Benjamini-Hochberg) < 0.05 were considered enriched. When using DAVID functional term clustering results, general themes were manually defined for significant term clusters (i.e., with scores > 1.3 as recommended by DAVID developers).

### Identification of differentially active regulatory circuits associated with the gain of tetracycline resistance.

We identified differentially active TFs using the NetSurgeon algorithm (41), as previously applied for Mycobacterium tuberculosis (92). Briefly, NetSurgeon ranks TFs based on their potential influence in the observed transcriptional changes between two states of interest (estimated according to the change in expression of their known target genes) (41). A total of 192 TF regulons were extracted from a transcriptional network (containing 5,517 signed TF-gene interactions) compiled from the RegulonDB version 9.0 database (40). Of the 192 TFs, 68 had less than 5 target genes and were not included in the analysis to reduce false positives due to overlap between regulons. We focused on the transcriptional changes between the Tet^R^ strain and the parental WT strain in the absence of tetracycline and the response of the Tet^R^ strain to tetracycline. TFs ranked (using the highest score between the independently computed scores for increased activity and decreased activity) in the top 15 of each comparison were considered differentially active. To complement the NetSurgeon analysis, a network component analysis (43) was applied to estimate the TF activity (TFA) using the transcriptional profile of their known targets. The RegulonDB-derived transcriptional network mentioned above and the microarray data reported by Händel et al. were used to estimate the TFA as previously described (32, 93). Statistical differences in TF activity were determined using a Welch’s *t* test. Only TFs with adjusted *P* values of <0.05 for basal and adaptive response that agreed with NetSurgeon-based predictions (Table 1) were considered differentially active. Finally, we mined the EGRIN model previously developed for E. coli (44) to identify clusters of coregulated genes statistically enriched (with adjusted hypergeometric test *P* values ≤ 0.05 and containing >9 DEGs) with genes whose expression was altered by gain of tetracycline resistance. The association between clusters with differentially expressed genes and TF regulons was used as an indicator of differential activity of the relevant TFs.

### Characterization of metabolic response of E. coli to drug treatment and *arcA* deletion.

We used the MetaboAnalyst 5.0 website (94) to analyze publicly available *Z*-score-normalized metabolic profiles of sertraline- and cefpiramide-treated E. coli (25) and *arcA* deletion E. coli (74). For each condition, the affected metabolites were defined as the ones within the highest 10% of absolute *Z* scores. To identify metabolically altered pathways due to drug treatments, we used the “pathway analysis” module available in the MetaboAnalyst platform using the hypergeometric test, relative-betweenness centrality, and the E. coli KEGG pathway library options. The input for this analysis was the KEGG IDs associated with perturbed metabolites. Only metabolic pathways with false discovery rate-adjusted hypergeometric test *P* values ≤ 0.25 were considered altered, following the threshold suggested by MetaboAnalyst developers. Similarly, we used the “joint-pathway analysis” module to identify metabolic pathways affected by the *arcA* deletion. This analysis integrated a list of DEGs due to *arcA* deletion (47) and a list of metabolites responding to *arcA* deletion (defined as described above) using the “metabolic pathways (integrated),” “hypergeometric test,” “relative-betweenness centrality,” and “combine queries” options for E. coli. Altered pathways were defined with the same adjusted *P* value threshold described above.

### Genome sequencing.

Late-log phase WT broth cultures were spun down, and the cells were lysed with lysis buffer (0.1% SDS, 0.1 M dithiothreitol [DTT], 10 mg/mL lysozyme in 0.1 M Tris-EDTA [TE] buffer). DNA was isolated from cell lysate using phenol-chloroform-isoamyl alcohol extraction method. Overnight Tet^R^ broth cultures were spun down. Cell pellets were resuspended in TE buffer. 10% SDS and proteinase K were added. DNA was precipitated with 100% ethanol added in a 3:1 volume ratio. The genomic DNA precipitate was washed with 70% ethanol and later dried out. Libraries for sequencing were prepared with the Nextera XT DNA library preparation kit (Illumina, San Diego, CA) for paired-end sequencing in a NextSeq instrument.

### Identification of mutations in the Tet^R^ strain.

Initial quality check and trimming of raw FASTQ files was performed using Trimmomatic 0.39 (95), with the following parameters: ILLUMINACLIP:NexteraPE-PE.fa:2:30:10, LEADING:3, TRAILING:3, SLIDINGWINDOW:4:15, and MINLEN:36. Reads that survived this filtering step were used for identifying mutations in the Tet^R^ strain, while taking into account background mutations present in the parental WT strain. Variant calling was performed with Snippy version 4.6.0 (https://github.com/tseemann/snippy) using default parameters and E. coli K-12 MG1655 genome (NC_000913.3) as a reference. Genomic coverage and identified variants are listed in Table S1.

### Genome-wide CRISPR KO library construction on Onyx.

Genome-wide KO libraries in WT and Tet^R^ background E. coli strains were generated on the Onyx Digital Genome Engineering Platform, a commercial benchtop instrument sold by Inscripta, Inc. Onyx (catalog number 1001176) is an automated platform that uses the MAD7 nuclease, a type V CRISPR nuclease from Eubacterium rectale, to generate multiplexed genome engineered libraries. All consumables, assays, and software used in this study are available at https://portal.inscriptacp.com/.

Compatibility of genome-wide libraries designed for WT E. coli with the Tet^R^ strain was confirmed by *de novo* genome assembly using short read polishing of Nanopore-based long reads using Raven (96) and Racon (97). Inscripta’s Onyx microbial strain analyzer (OMSA) tool was used to confirm that 99.9% of designs in the library are predicted to function in the Tet^R^ strain background. The genome-wide KO library included 8,271 intended edits representing approximately two deletion mutants per gene: a triple-stop (TAATAATAA) substitution at amino acid position 10 and a triple-stop insertion at amino acid position 15.

Single E. coli WT or Tet^R^ colonies were isolated from an LB agar plate and grown overnight in LB to saturation and diluted to optical density at 600 nm (OD_600_) of 2.5 before subsequent processing. 1 mL of cell suspension was subsequently prepared using the Onyx E. coli edit competency kit (GEN-EC-1004). 1 mL of E. coli cells (approximately 6 × 10^8^ cells) prepared using the edit competency kit were placed into the Onyx instrument. The OnyxWare program K-strain version 1.1 was selected, and the Onyx run was initiated. Briefly, the instrument transferred the cells to a cell growth cuvette (reference number 1001155/catalog number GEN-EC-1007) for growth to 0.5 OD_600_, as measured on the instrument. After an initial outgrowth, the instrument transferred cells to the microfluidic cell controller (reference number 1001152/catalog number GEN-EC-1007). There, the cells were prepared for electroporation using media exchange. Once they were rendered competent, the instrument moved the cells to the microfluidic cell transformer (reference number 1001152/catalog number GEN-EC-1007), which controls introduction of the MAD7-containing “engine” plasmid, as well as the gRNA/repair template/barcode-containing plasmid into cells by electroporation. Following electroporation, the cells were placed by the instrument into a second cell growth cuvette (reference number 1002161/catalog number GEN-EC-1007) for recovery. The cells were then transferred to the digital engineering processor (reference number 1001153/catalog number GEN-EC-1007) for abundance normalization. The resulting normalized pool of cells was collected as multiple tubes from the instrument. Per library, 5 mL of cells were collected at an OD_600_ ranging from 3.2 to 3.7. The cells were immediately stored frozen at −80°C in 15.5% glycerol. Depending on cell growth, the total run time on the instrument for E. coli lasted around 48 h.

Following editing, the pooled libraries were grown off-instrument for approximately 8 h in TB supplemented with 1,000 μg/mL carbenicillin and 68 μg/mL chloramphenicol. Library edit fractions were estimated using pooled whole-genome sequencing (pWGS) and ranged from 45.3 to 50.8% (98). Based on statistical approaches described by Cawley et al. (98), we expect to observe 96.9 to 97.1% of all designs in selections using these libraries, assuming the selections start with ≥1 × 10^6^ cells.

### Competitive assays of pooled mutant libraries.

To evaluate the impact of single-gene deletions in the fitness of the Tet^R^ strain, we performed broth macrodilution growth experiments as described before. Notably, instead of using overnight cultures as inocula, we grew five aliquots (each with 0.2 mL, previously stored at −80°C) of the relevant library in LB with 99.9 μg/mL of carbenicillin for 4 to 6 h (we refer to these cultures as the library acclimatization cultures, or “t0”). Carbenicillin was added along all experiments to maintain the plasmid harboring DNA barcodes for each gene edit. For each library, four acclimatization cultures were used to start four 0.1 OD_600_ 10-mL LB cultures with tetracycline (0.75 μg/mL for WT libraries and 20 μg/mL for Tet^R^ libraries) and without tetracycline (i.e., adding ethanol as a control as previously explained). Sub-MIC tetracycline concentrations were selected as explained before. Pooled library cultures were maintained in mid-log phase during three growth cycles (i.e., from 0.1 OD_600_ to ~1.0 OD_600_). At the end of each growth cycle (i.e., when cultures were in the 0.8 to 1.2 OD_600_ interval), culture aliquots were used to create cell pellets stored at −80°C. Cell pellets of the acclimatization cultures were also stored. As in previous experiments, two control LB cultures were included in each experiment.

### Barcode sequencing fitness estimation.

Cell pellets from the competition assays described in the previous section were used for DNA extraction following Inscripta protocol with the Wizard SV genomic DNA purification system (Promega). DNA libraries of previously extracted DNA were prepared using the 48-sample Onyx barcode diversity assay kit (Inscripta, Boulder, CO) following the manufacturer’s instructions. Four available replicates of each analyzed mutant library were sequenced. Prepared libraries were sequenced (as single-end 100-bp reads) in a NextSeq instrument. An average of 972 (± 321) reads per design for each replicate was observed.

Raw sequencing data were processed on Illumina BaseSpace suite. The resulting FASTQ files were processed with the InscriptaResolver software to estimate read counts for each gene deletion (Data Set S1). We used the number of reads for each unique gene edit barcode sequence as a proxy for mutant frequency in the genome-wide single-gene deletion competition assays in order to identify mutants over- and under-represented in the population. In this way, we were able to identify mutations that were deleterious (under-represented) or beneficial (over-represented). The ALDEx2 R package (51) was used to identify differentially abundant gene deletion designs. Briefly, we used ALDEx2 interquartile log ratio transformation to estimate relative abundance of each mutant with 1,000 Monte Carlo instances. Before each comparison, we removed all deletion mutants with less than 10 reads in all four replicates (referred to as ‘dropouts’) of any of the two time points being compared. We define differentially abundant mutants at ‘t1’ and ‘t2’ with respect to ‘t0’ as those constructs with Benjamini-Hochberg adjusted (Welch’s and Wilcoxon) *t* test *P* values < 0.1 and absolute ALDEx2-estimated effect >2, as suggested by ALDEx2 developers and others (99, 100). Changes in abundance for each KO design at each time point (with respect to t0) correspond to ALDEx2-computed “diff.btw” scores. Data for the last time point (t3) was not used in this analysis due to the high number of depleted mutants at the end of the experiment (due to tetracycline selection pressure) (Fig. 3A) and the challenge this may represent for ALDEx2 posterior relative abundance estimation (51). To focus on the genes most likely affecting fitness, a gene was only considered to affect fitness if all of its deletion mutants (excluding any dropout) were differentially abundant and had the same effect (deleterious or beneficial).

### Code availability.

An R notebook (101) with all necessary scripts and input files to generate all figures of this article are publicly available in the following GitHub repository: https://github.com/marioluisao/Compensatory-mechanisms-for-Antimicrobial-Resistance.

### Materials availability.

Material requests may be directed to the corresponding author, Nitin S. Baliga (nitin.baliga@isbscience.org).

### Data availability.

The data generated in this study are available in the following GitHub repository: https://github.com/marioluisao/Compensatory-mechanisms-for-Antimicrobial-Resistance.
