# Supplementary material for: Disrupting the ArcA Regulatory Network Amplifies the Fitness Cost of Tetracycline Resistance in Escherichia coli
Source: mSystems. 2022 Dec 20;8(1):e00904-22. doi: 10.1128/msystems.00904-22 (PMC9948699; doi:10.1128/msystems.00904-22)
Supplement: TABLE S1 [file msystems.00904-22-s0007.docx]

**Table S1. Mutations identified in the Tet^R^ strain.**

| **Strain** | **Number of reads*^a^*** | **Average depth of coverage*^b^*** | **Gene*^c^*** | **Genomic coordinate** | **Ref. 🡺 variant*^d,e^*** | **Variant annotation*^d^*** | **Variant frequency^f^** |
| --- | --- | --- | --- | --- | --- | --- | --- |
| Tet^R^ | 1,419,018 | 23.0 | *acrB* | 484271 | T 🡺 G | Missense | 1.00 (30/30) |
|  |  |  | *acrR* | 486014 | C 🡺 A | Missense | 1.00 (33/33) |
|  |  |  | *ompF* | 986771 | G 🡺 GTTTCCCC | Frameshift | 0.94 (16/17) |
|  |  |  | *gatC****^g^*** | 2173360 | ACC 🡺 A | Frameshift | 1.00 (22/22) |
|  |  |  | *mlaA* | 2464873 | ATTGAAG 🡺 A | In-frame deletion | 1.00 (12/12) |
|  |  |  | *glpR****^g^*** | 3560455 | C 🡺 CG | Frameshift | 1.00 (27/27) |
| WT | 4,626,697 | 71.8 | *gatC* | 2173360 | ACC 🡺 A | Frameshift | 1.00 (44/44) |
|  |  |  | *eutJ* | 2570086 | C 🡺 T | Synonymous | 1.00 (120/120) |
|  |  |  | *glpR* | 3560455 | C 🡺 CG | Frameshift | 0.99 (106/107) |

*^a^*Total number of reads in the BAM alignment file generated by the variant calling tool Snippy (https://github.com/tseemann/snippy).

^b^Estimated using SAMtools (Danecek P, Bonfield JK, Liddle J, Marshall J, et al., Gigascience 10:giab008, 2021) and the BAM file generated by Snippy.

*^c^*Mutated gene.

*^d^*Mutations identified using Snippy.

*^e^*Mutations are indicated with the arrow symbol (“🡺”). Reference sequence and detected variants are shown before and after the arrow, respectively.

*^f^*Frequency was defined as the number of reads with the identified variant divided by the total number of reads mapped to the genomic region of interest (i.e., reads that match the sequence in the refence genome and reads with the identified mutation). Both values are shown in parenthesis.

*^g^*Mutations already present in the parental susceptible WT strain.
