# Supplementary figures and images for: Disrupting the ArcA Regulatory Network Amplifies the Fitness Cost of Tetracycline Resistance in Escherichia coli
Source: mSystems. 2022 Dec 20;8(1):e00904-22. doi: 10.1128/msystems.00904-22 (PMC9948699; doi:10.1128/msystems.00904-22)

Log2 fold-change  
(vs. WT no antibiotic)

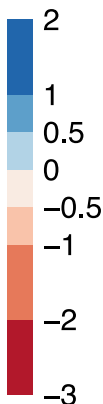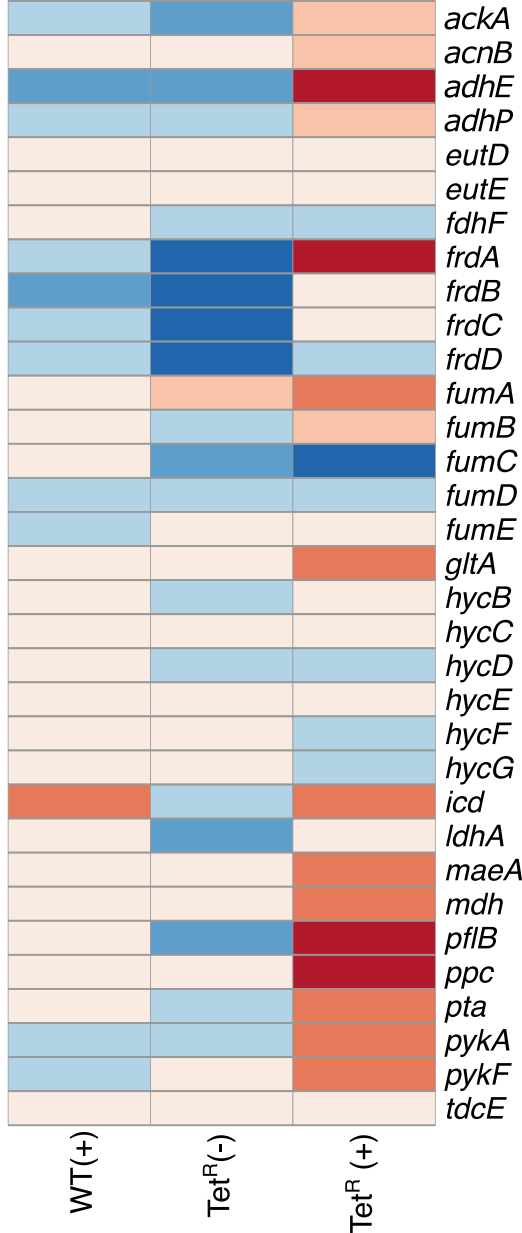

Supplement: FIG S1 [file msystems.00904-22-s0001.pdf]

**A**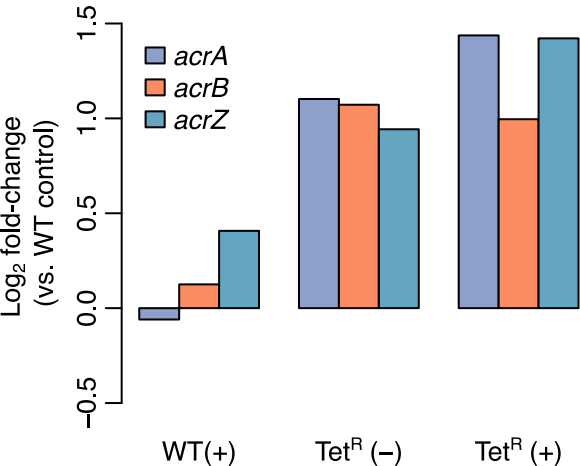**B**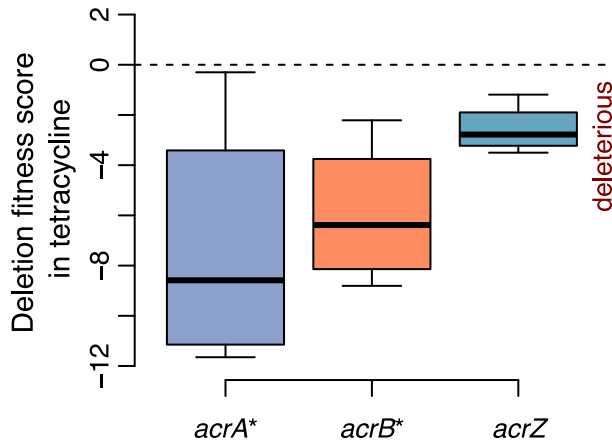

Supplement: FIG S2 [file msystems.00904-22-s0002.pdf]

A

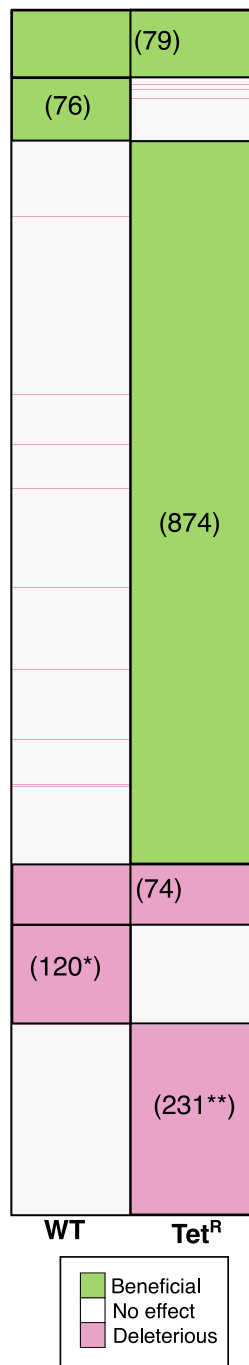

C

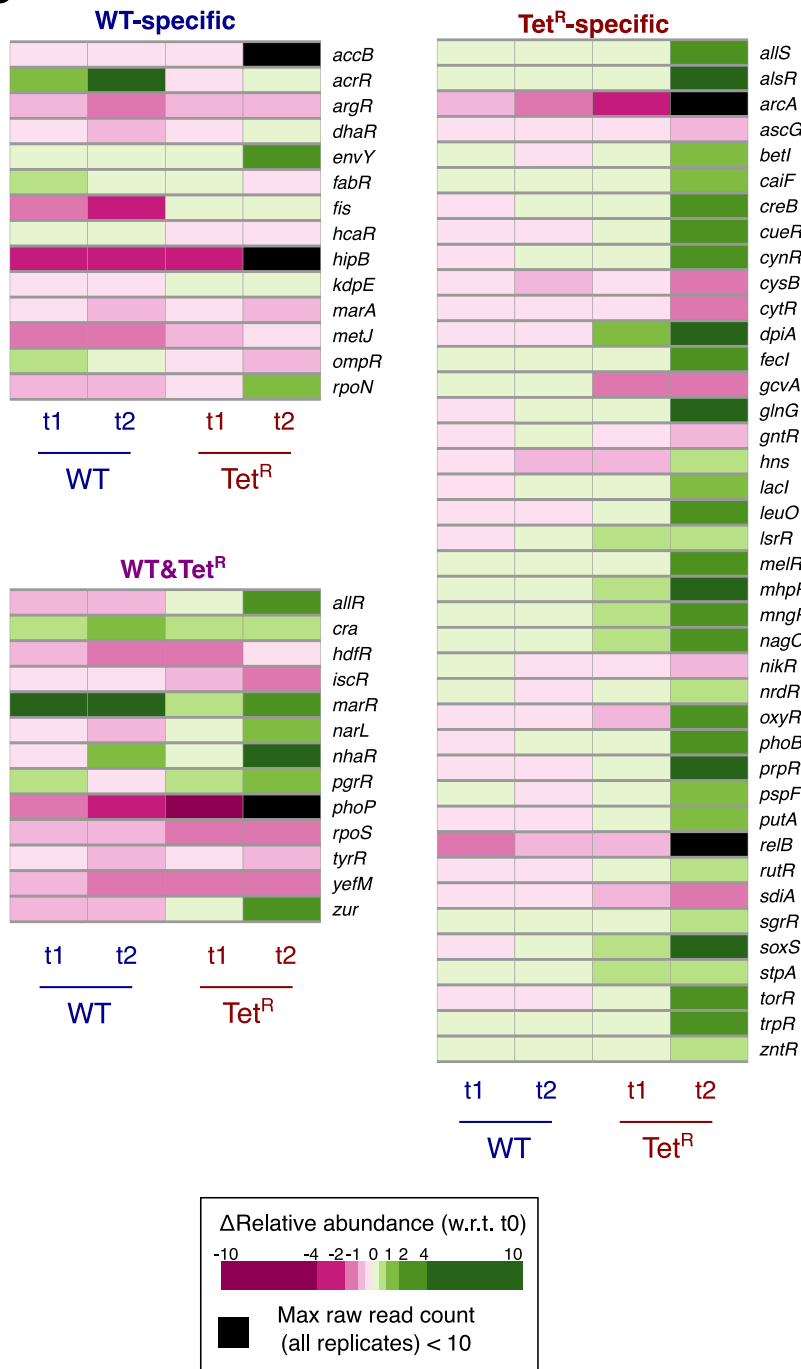

B

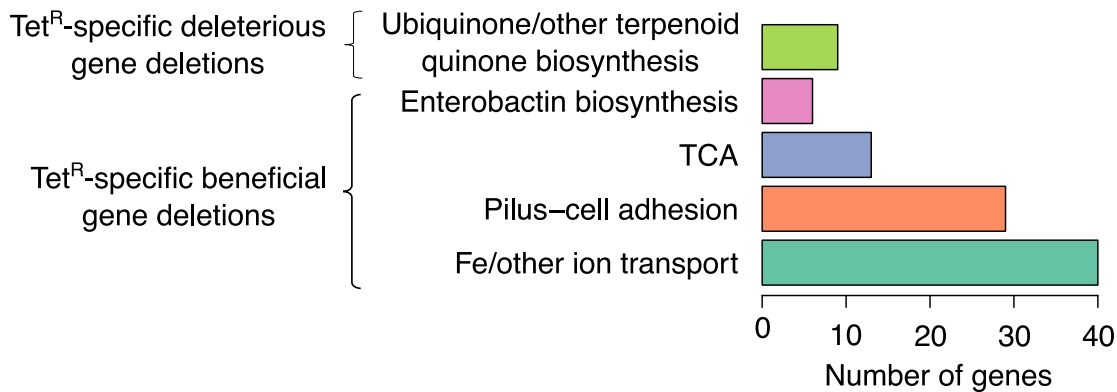

Supplement: FIG S3 [file msystems.00904-22-s0003.pdf]

**A***menB* (6419317)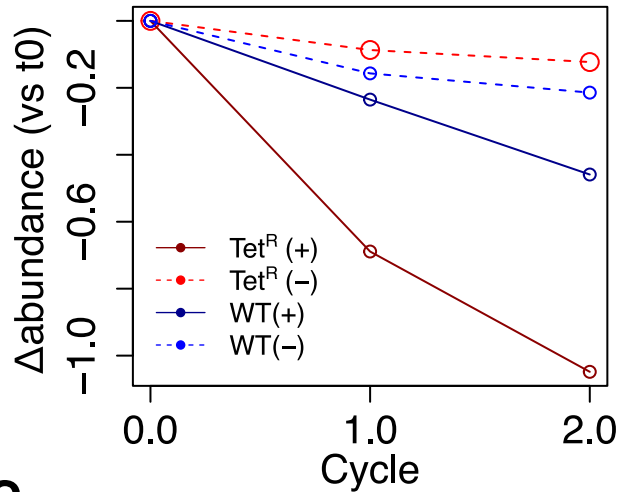**B***frdB* (6416702)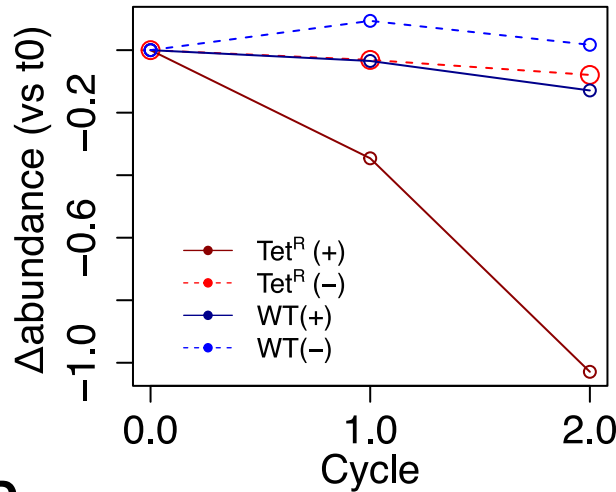**C***hybB* (6418292)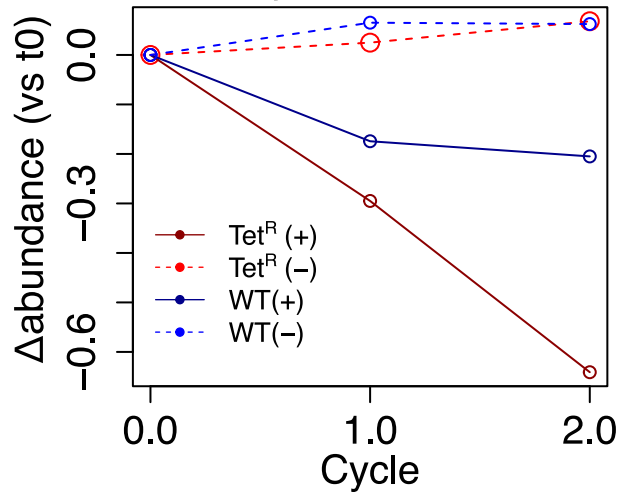**D***mdh* (6419091)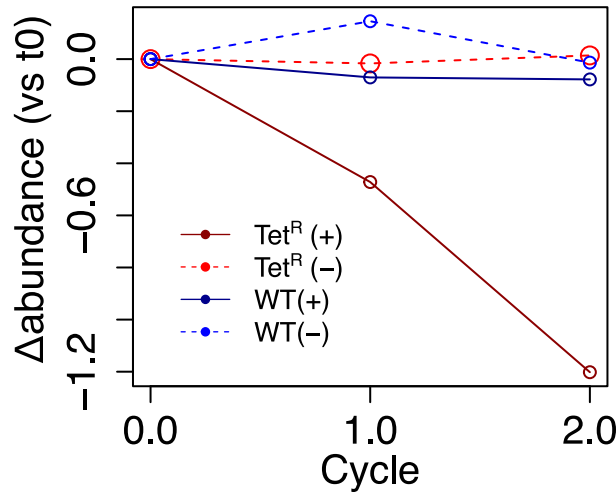

Supplement: FIG S4 [file msystems.00904-22-s0004.pdf]

**A**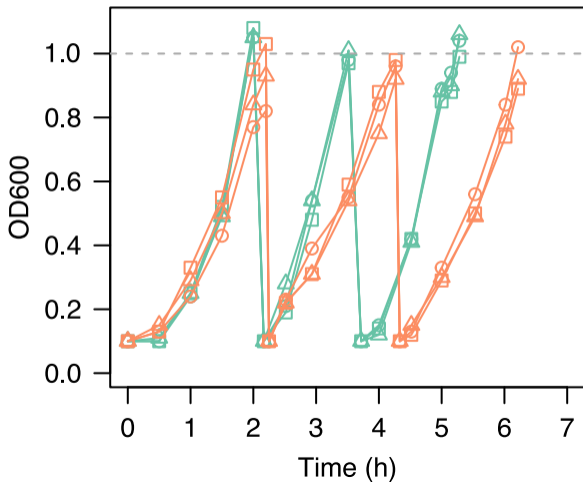**B**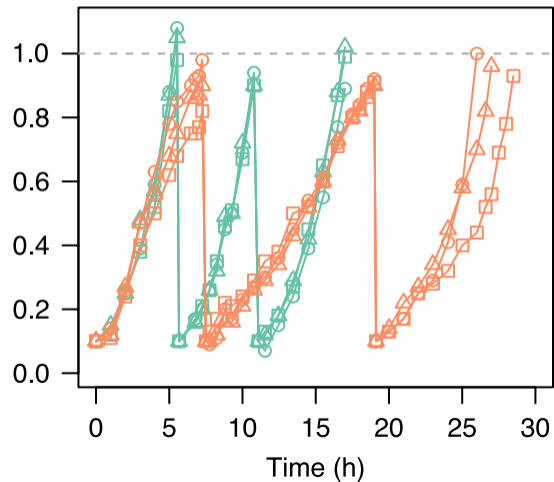

Supplement: FIG S5 [file msystems.00904-22-s0005.pdf]

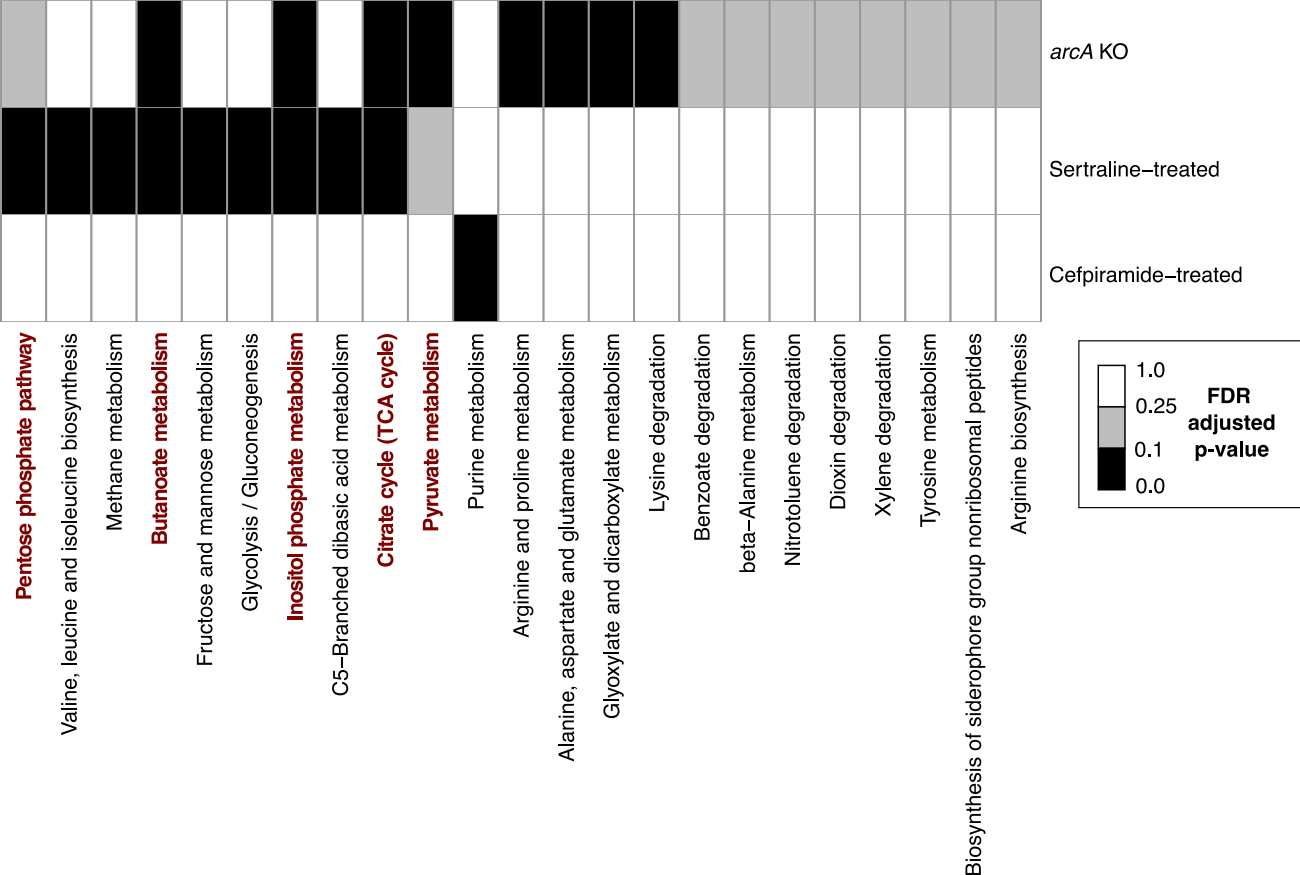

Supplement: FIG S6 [file msystems.00904-22-s0006.pdf]
