# Supplementary material for: Disrupting the ArcA Regulatory Network Amplifies the Fitness Cost of Tetracycline Resistance in Escherichia coli
Source: mSystems. 2022 Dec 20;8(1):e00904-22. doi: 10.1128/msystems.00904-22 (PMC9948699; doi:10.1128/msystems.00904-22)
Supplement: TABLE S2 [file msystems.00904-22-s0008.docx]

**Table S2. Functional enrichment information of genes differentially expressed in the WT and Tet^R^ strains.**

| **Functional term*^a^*** | **WT**  (+TET)*^b^* | **Tet^R^** | | **Total number of genes associated with term** | **P-value** |
| --- | --- | --- | --- | --- | --- |
|  |  | (-TET) *^b^* | (+TET) *^b^* |  |  |
|  | DEGs*^c,d^* | DEGs*^c,d^* | DEGs*^c,d^* |  |  |
| GO:0015803~branched-chain amino acid transport | 5 | - | - | 7 | 1.97E-06 |
| GO:0006865~amino acid transport | 8 | - | - | 87 | 1.09E-05 |
| GO:0098713~leucine import across plasma membrane | 4 | - | - | 4 | 1.52E-05 |
| KW-0029~Amino-acid transport | 8 | - | - | 79 | 2.35E-05 |
| GO:0015658~branched-chain amino acid transmembrane transporter activity | 4 | - | - | 5 | 3.11E-05 |
| GO:0005304~L-valine transmembrane transporter activity | 4 | - | - | 5 | 3.11E-05 |
| GO:0015188~L-isoleucine transmembrane transporter activity | 4 | - | - | 5 | 3.11E-05 |
| GO:1903714~isoleucine transmembrane transport | 4 | - | - | 5 | 2.83E-05 |
| eco02024:Quorum sensing | 7 | - | - | 64 | 6.71E-05 |
| GO:0015190~L-leucine transmembrane transporter activity | 4 | - | - | 6 | 4.65E-05 |
| GO:0015823~phenylalanine transport | 4 | - | - | 6 | 4.52E-05 |
| GO:1903785~L-valine transmembrane transport | 4 | - | - | 8 | 0.0001 |
| GO:0055052~ATP-binding cassette (ABC) transporter complex, substrate-binding subunit-containing | 5 | - | - | 34 | 0.0015 |
| GO:0015192~L-phenylalanine transmembrane transporter activity | 3 | - | - | 4 | 0.0022 |
| eco02010:ABC transporters | 7 | - | - | 179 | 0.0123 |
| KW-0813~Transport | 13 | - | - | 732 | 0.0339 |
| KW-0346~Stress response | 7 | 19 | 38 | 150 | 0.0152;  3.54e-07;  2.96e-08 |
| KW-0805~Transcription regulation | 9 | - | 53 | 337 | 0.0202;  2e-04 |
| KW-0804~Transcription | 9 | - | 53 | 343 | 0.0202;  2e-04 |
| KW-0732~Signal | 15 | - | - | 479 | 0.0197 |
| KW-0658~Purine biosynthesis | - | 6 | 8 | 17 | 7e-04;  0.0423 |
| GO:0006164~purine nucleotide biosynthetic process | - | 6 | - | 18 | 0.0033 |
| GO:0006189~'de novo' IMP biosynthetic process | - | 5 | - | 12 | 0.0054 |
| eco01110:Biosynthesis of secondary metabolites | - | 20 | 130 | 339 | 0.0054;  1.24e-21 |
| GO:0044205~'de novo' UMP biosynthetic process | - | 4 | - | 7 | 0.0137 |
| eco00230:Purine metabolism | - | 9 | - | 78 | 0.0090 |
| GO:0006207~'de novo' pyrimidine nucleobase biosynthetic process | - | 4 | - | 9 | 0.0240 |
| KW-0210~Decarboxylase | - | 5 | - | 21 | 0.0279 |
| GO:0006221~pyrimidine nucleotide biosynthetic process | - | 4 | - | 11 | 0.0365 |
| KW-0665~Pyrimidine biosynthesis | - | 4 | 7 | 11 | 0.0248;  0.0169 |
| eco00240:Pyrimidine metabolism | - | 7 | - | 57 | 0.0301 |
| KW-1134~Transmembrane beta strand | - | 6 | - | 45 | 0.0258 |
| eco01230:Biosynthesis of amino acids | - | - | 67 | 117 | 9.12E-21 |
| KW-0028~Amino-acid biosynthesis | - | - | 52 | 104 | 3.82E-18 |
| GO:0008652~cellular amino acid biosynthetic process | - | - | 52 | 103 | 3.04E-17 |
| eco01100:Metabolic pathways | - | - | 235 | 917 | 1.70E-17 |
| GO:0003824~catalytic activity | - | - | 93 | 375 | 5.14E-08 |
| eco01200:Carbon metabolism | - | - | 48 | 110 | 8.82E-09 |
| eco00250:Alanine, aspartate and glutamate metabolism | - | - | 22 | 33 | 1.16E-07 |
| KW-0816~Tricarboxylic acid cycle | - | - | 16 | 23 | 3.63E-07 |
| GO:0006099~tricarboxylic acid cycle | - | - | 18 | 29 | 2.20E-06 |
| GO:0005829~cytosol | - | - | 194 | 1051 | 2.54E-06 |
| KW-0007~Acetylation | - | - | 40 | 100 | 5.96E-07 |
| eco01120:Microbial metabolism in diverse environments | - | - | 82 | 268 | 1.05E-06 |
| KW-0055~Arginine biosynthesis | - | - | 11 | 12 | 2.22E-06 |
| GO:0006526~arginine biosynthetic process | - | - | 11 | 12 | 1.28E-05 |
| GO:0042802~identical protein binding | - | - | 97 | 456 | 5.87E-05 |
| eco00190:Oxidative phosphorylation | - | - | 23 | 43 | 8.46E-06 |
| GO:0009060~aerobic respiration | - | - | 14 | 25 | 0.0004 |
| GO:0006096~glycolytic process | - | - | 12 | 19 | 0.0005 |
| eco01210:2-Oxocarboxylic acid metabolism | - | - | 16 | 26 | 7.35E-05 |
| eco00220:Arginine biosynthesis | - | - | 13 | 18 | 7.35E-05 |
| BINDING:Substrate | - | - | 58 | 259 | 0.0225 |
| KW-0324~Glycolysis | - | - | 11 | 18 | 0.0005 |
| GO:0016491~oxidoreductase activity | - | - | 74 | 356 | 0.0044 |
| GO:0006541~glutamine metabolic process | - | - | 10 | 15 | 0.0023 |
| eco00650:Butanoate metabolism | - | - | 18 | 35 | 0.0003 |
| eco00340:Histidine metabolism | - | - | 8 | 8 | 0.0004 |
| KW-0368~Histidine biosynthesis | - | - | 8 | 10 | 0.0011 |
| GO:0000105~histidine biosynthetic process | - | - | 8 | 10 | 0.0043 |
| eco00010:Glycolysis / Gluconeogenesis | - | - | 21 | 47 | 0.0005 |
| ACT_SITE:Proton donor | - | - | 35 | 135 | 0.0397 |
| GO:0030170~pyridoxal phosphate binding | - | - | 20 | 58 | 0.0122 |
| KW-0315~Glutamine amidotransferase | - | - | 8 | 12 | 0.0011 |
| GO:0006974~cellular response to DNA damage stimulus | - | - | 54 | 250 | 0.0084 |
| eco00020:Citrate cycle (TCA cycle) | - | - | 15 | 29 | 0.0013 |
| GO:0051287~NAD binding | - | - | 16 | 43 | 0.0255 |
| GO:0006094~gluconeogenesis | - | - | 9 | 15 | 0.0141 |
| GO:0006520~cellular amino acid metabolic process | - | - | 12 | 28 | 0.0251 |
| GO:0006807~nitrogen compound metabolic process | - | - | 10 | 21 | 0.0365 |
| eco00620:Pyruvate metabolism | - | - | 22 | 59 | 0.0060 |
| eco00680:Methane metabolism | - | - | 15 | 33 | 0.0060 |
| KW-0100~Branched-chain amino acid biosynthesis | - | - | 9 | 18 | 0.0169 |
| KW-0830~Ubiquinone | - | - | 9 | 15 | 0.0368 |
| GO:0030964~NADH dehydrogenase complex | - | - | 8 | 14 | 0.0465 |
| KW-0298~Galactitol metabolism | - | - | 5 | 5 | 0.0169 |
| KW-0874~Quinone | - | - | 9 | 14 | 0.0067 |
| KW-0663~Pyridoxal phosphate | - | - | 20 | 59 | 0.0368 |
| eco00500:Starch and sucrose metabolism | - | - | 15 | 36 | 0.0154 |
| eco00260:Glycine, serine and threonine metabolism | - | - | 15 | 38 | 0.0261 |
| eco00030:Pentose phosphate pathway | - | - | 13 | 32 | 0.0379 |
| eco00400:Phenylalanine, tyrosine and tryptophan biosynthesis | - | - | 10 | 21 | 0.0379 |
| eco00310:Lysine degradation | - | - | 9 | 18 | 0.0435 |
| KW-1277~Toxin-antitoxin system | - | - | 22 | 52 | 2.19E-08 |
| KW-0238~DNA-binding | - | - | 65 | 439 | 2.06E-05 |
| GO:0003677~DNA binding | - | - | 73 | 499 | 0.0003 |
| eco00540:Lipopolysaccharide biosynthesis | - | - | 11 | 38 | 0.0005 |
| KW-0281~Fimbrium | - | - | 10 | 28 | 0.0024 |
| KW-0229~DNA integration | - | - | 7 | 14 | 0.0090 |
| KW-0255~Endonuclease | - | - | 12 | 45 | 0.0128 |
| KW-0678~Repressor | - | - | 25 | 150 | 0.0128 |
| KW-0448~Lipopolysaccharide biosynthesis | - | - | 14 | 65 | 0.0346 |
| KW-0540~Nuclease | - | - | 14 | 70 | 0.0408 |

*^a^*Enrichment analyses were performed using DAVID (Huang DW, Sherman BT, Lempicki RA, Nat Protoc 4:44., 2008). Only terms with adjusted p-values (Benjamini-Hochberg) < 0.05 were considered enriched.

*^b^*Presence and absence of tetracycline is indicated as ‘(+TET)’ and ‘(-TET)’.

*^c^*Number of significantly up- and down-regulated genes (DEGs) with respect to the WT strain in antibiotic free condition were independently analyzed.

*^d^*Terms associated with up- and down-regulated genes are shown in blue and red boxes, respectively. Only the number of DEGs associated with over-represented terms are shown.
